# Sex-dependent effects of a high-fat diet on the hypothalamic response in mice

**DOI:** 10.1186/s13293-025-00699-3

**Published:** 2025-02-25

**Authors:** Virginie Dreux, Candice Lefebvre, Charles-Edward Breemeersch, Colin Salaün, Christine Bôle-Feysot, Charlène Guérin, Pierre Déchelotte, Alexis Goichon, Moïse Coëffier, Ludovic Langlois

**Affiliations:** 1 Univ Rouen Normandie, INSERM, Normandie Univ, ADEN UMR1073 “Nutrition, Inflammation and Microbiota-Gut-Brain Axis, F-76000 Rouen, France; 2 Univ Rouen Normandie, Institute for Research and Innovation in Biomedicine (IRIB), F-76000 Rouen, France; 3 Department of Nutrition, CHU Rouen, F-76000 Rouen, France

**Keywords:** Obesity, High fat diet, Hypothalamus, Arcuate nucleus, Neuroinflammation, Microglia, Astrocytes

## Abstract

Sex differences in rodent models of diet-induced obesity are still poorly documented, particularly regarding how central mechanisms vary between sexes in response to an obesogenic diet. Here, we wanted to determine whether obese phenotype and hypothalamic response to a high-fat diet (HFD) differed between male and female C57Bl/6J mice. Mice were exposed to either a 60% HFD or a standard diet first for both a long- (14 weeks) and shorter-periods of time (3, 7, 14 and 28 days). Analysis of the expression profile of key neuronal, glial and inflammatory hypothalamic markers was performed using RT-qPCR. In addition, astrocytic and microglial morphology was examined in the arcuate nucleus. Monitoring of body weight and composition revealed that body weight and fat mass gain appeared earlier and was more pronounced in male mice. After 14 weeks of HFD exposure, normalized increase of body weight reached similar levels between male and female mice. Overall, both sexes under HFD displayed a decrease of orexigenic neuropeptides expression while an increase in *Pomc* gene expression was observed only in female mice. In addition, changes in the expression of hypothalamic inflammatory markers were relatively modest. We also reported that the glial cell markers expression and morphology were affected by HFD in a sex- and time dependent manner, suggesting a more pronounced glial cell activation in female mice. Taken together, these data show that male and female mice responded differently to HFD exposure, both on short- and long-term and suggest that a strong inflammatory hypothalamic profile is not systematically present in diet-induced obesity models. Nevertheless, in addition to these present data, the underlying mechanisms should be deciphered in further investigations.

**Supplementary Information:**

The online version contains supplementary material available at 10.1186/s13293-025-00699-3.

## Abstract (plain language summary)

This study looked at how male and female mice respond differently to a high-fat diet (HFD), particularly focusing on changes in the brain’s hypothalamus, which is involved in regulating hunger and body weight. A common mouse strain was used and exposed to either HFD or a standard diet for various lengths of time (ranging from 3 days to 14 weeks). Male mice gained weight and fat earlier and more significantly than females. However, after 14 weeks, both sexes had similar overall weight gain. In the brain, both males and females showed a reduction in hunger-stimulating neuropeptides. Interestingly, only female mice showed an increase in *Pomc* gene expression, which is associated with reducing food intake. Inflammatory responses in the hypothalamus were mild overall, and the study found that the morphology of certain brain cells (astrocytes and microglia) differed between males and females. This suggests that males and females react differently to a HFD, both in the short- and long-term, and that obesity in these models does not always involve a strong inflammatory response in the hypothalamus.

**Supplementary Information:**

The online version contains supplementary material available at 10.1186/s13293-025-00699-3.

## Highlights

- Male mice are more vulnerable to HFD at earlier stages of diet exposure
- HFD induces a dysregulation of neuropeptides expression in the hypothalamus
- The presence of hypothalamic inflammation is not systematic in DIO model
- Glial cells exhibit sex-dependent structural remodeling under HFD feeding
- Contrarily to astrocytes, HFD-fed mice showed lower microglial complexity

**Supplementary Information:**

The online version contains supplementary material available at 10.1186/s13293-025-00699-3.

## Background

The World Health Organization reported that the prevalence of obesity has been rising worldwide for several decades reaching 13% of adults (11% of men and 15% of women) in 2016. Obesity is associated with many comorbidities (cardiovascular diseases, metabolic syndrome, anxio-depressive disorders), thus representing a major public health problem [49]. This multifactorial disease is characterized by an energy imbalance mostly due to changes in the dietary and physical activity patterns of the modern population. In particular, the consumption of energy-dense foods and excess lipids leads to low-grade chronic inflammation, the principal component of obesity [17].

In periphery, this inflammatory response is orchestrated by metabolic organs (including adipose tissue, liver, muscle and pancreas) and involves several pathways [17]. One of them is related to the expansion and hypertrophy of adipocytes, which release proinflammatory cytokines and free fatty acids in systemic circulation, activating the immune system [39]. The role of gut microbiota is also discussed via microbiota-gut-brain communication [43]. The inflammatory state disrupts especially the hypothalamic function [12, 47].

Given the critical role of the hypothalamus in appetite control and energy expenditure [28, 41]), extensive studies have been carried out to examine the link between hypothalamic inflammation and the pathophysiology of obesity and associated metabolic disorders [3, 6, 24, 46]. These studies demonstrated that the diet-related brain inflammatory response precedes weight gain or obesity development [47]. Indeed, hypothalamic inflammatory response can be detected at the postprandial time scale [7]. Although the underlying biological processes and mediators involved still remain unclear, glial cells such as microglia and astrocytes surrounding neurons located in brain areas linked to food intake regulation have been proposed as major cellular contributors to hypothalamic inflammation [25, 37]. Especially, a reactive gliosis was described in response to the consumption of HFD. This glial signature is characterized by morphological and phenotypic changes (increased ionized calcium-binding adapter molecule 1, Iba1 and glial fibrillary acidic protein, GFAP expressions) associated with functional dysregulation [25, 37, 42, 50].

Despite the female predominance in obesity, most of preclinical studies have been conducted in male rodents [1, 27, 35]. Nevertheless, some data reported sex differences, with females exhibiting an overall resistance to the obesogenic effects of HFD [23, 26, 29]. In addition, research has been focused on the effects of either long-term or short-term exposure to HFD, and often in other brain structures such as the hippocampus [11], the ventral tegmental area [31] or the nucleus accumbens [13]. Consequently, sex differences in the hypothalamic response after different time exposure to HFD remain poorly documented within the same study.

In the present study, we aimed to evaluate how sex and the duration of exposure to HFD can influence the characteristics of the diet-induced obesity model (DIO). We investigated the hypothalamic response after long- and short-term exposure to HFD in C57BL/6J male and female mice, focusing on the assessment of expression levels of several inflammatory markers and glial cell-specific markers.

## Materials and methods

### Animal experimentation

Six-week-old C57Bl/6J male and female mice were obtained from Janvier Labs (Le Genest-Saint-Isle, France, n = 24/sex, experiment 1; n = 72/sex, experiment 2). Mice were socially housed (n = 4/cage) in a controlled environment (20 ± 2 °C with a 12-h light/dark cycle) with free access to food and water. After 1 week of acclimatization, male and female mice were randomized into different groups receiving for 14 weeks (experiment 1, w14) or for 3, 7, 14, and 28 days (experiment 2, d3, d7, d14, d28) either a standard diet providing 14% from fat, 27% from proteins and 59% from carbohydrates (SD, 3.34 kcal/g, 1314 formula, Altromin, Lage, Germany) or high fat diet (HFD) providing 60% kcal as fat, 20% kcal as carbohydrates and 20% kcal as protein (5.24 kcal/g, D12492i, Research Diet, New Brunswick, NJ, US, Supplementary Table S1). Body weight was monitored weekly, and body composition was assessed by EchoMRI (EchoMRI, Houston, TX, US) at the end of each time of diet exposure. The weekly food intake was measured in the beginning of light cycle and the animals were euthanized two hours after the beginning of light cycle. All animal experiments were carried out in accordance with ARRIVE guidelines and the EU Directive 2010/63/EU for animal experiments. The protocols used were approved by the regional ethics committee and authorized by the French Ministry of Higher Education, Research and Innovation (authorization on APAFIS #29283–2021012114574889 v5).

### Tissue sampling

Prior to tissue collection, mice were deeply anesthetized by intraperitoneal injection of a ketamine/xylazine solution (100 and 10 mg/kg, respectively). Blood samples were collected, centrifuged (3000* g,* 4 °C for 15 min) in heparinized tubes and the plasma was frozen at − 80 °C. Mice were decapitated and the brains were removed on ice either for immunohistochemistry analyses or for hypothalamus dissection. The brain and hypothalamus were immediately placed in a 4% paraformaldehyde (PFA) buffered solution or frozen in liquid nitrogen and stored at − 80 °C, respectively.

### Brain slice preparation and immunofluorescence

The whole brains (n = 16 for experiment 1; n = 72 for experiment 2) were post-fixed for 24 h in 4% PFA buffered solution at room temperature (RT), then cryoprotected into a 30% sucrose solution (in 0.1 M phosphate-buffered saline (PBS)). Following at least 24 h of cryoprotection, the brains were frozen between − 20 and − 30 °C in isopentane before being stored at − 80 °C. Serial coronal Sections (20 µm-thick), located − 1.22 to − 2.54 mm from bregma based on brain atlas coordinates (The Mouse Brain in Stereotaxic Coordinates, 3rd Edition, Franklin and Paxinos, 2008), were cut using a CM 1950 cryostat (Leica Biosystems). Sections were mounted on Superfrost Plus microscope slides (Thermo Fisher Scientific, Portmouth, US) and then stored at − 20 °C.

Before immunohistochemistry, the slides were rehydrated in PBS (15 min). Then, heat-induced epitope retrieval was performed using 10 mM sodium citrate (pH 8.5) buffer for 20 min at 80 °C. Next, brain slices were blocked in 5% bovine serum albumin (BSA) (Eurobio Abcys, Courtaboeuf, France) in PBS for 30 min at RT and incubated overnight at 4 °C with the following antibodies: rat GFAP monoclonal immunoglobulin G (IgG, Cat. No #13–0300, 1:500, Invitrogen, Rockford, US), and rabbit IBA1 polyclonal IgG (Cat. No #019–19741, 1:2000, FUJIFILM Wako Pure Chemical, Osaka, Japan) in PBS, 0.3% Triton X-100, 0.01% NaN_3_. The next day, after being washed in PBS (3 × 10 min) and TNT solution (1.2% Tris; 0.9% NaCl; 1 mL Tween 20; distilled water q.s. 2 L) (30 min), the slides were blocked with TNB solution for 30 min (1.2% Tris; 0.9% NaCl; 0.01% skim milk in distilled water) and incubated with Alexa Fluor 488-conjugated anti-rat IgG secondary antibody (Cat. No #A11006, 1:400, Invitrogen,) and Alexa Fluor 555-conjugated anti-rabbit IgG secondary antibody (Cat. No # A21428,1:400; Life Technologies,) protected from light for 2 h. After washing in TNT (3 × 10 min), the slides sections were mounted between the slide and coverslip with a solution of Fluoroshield with DAPI (Sigma Aldrich, Saint Louis, MO, US) and then stored at 4 °C in the dark until microscopic observation. The coverslips were sealed with nail polish to prevent desiccation and movement of the samples under the microscope. Controls without primary antibodies were used to check the absence of nonspecific coupling of secondary antibodies.

### Image acquisition

#### Cell counting

For the experiment 1, images (1936 × 1460 pixels, pixel size 0.23 µm) were obtained using APOTOME ZEISS Axioimager Z1 fluorescence microscope. Z-stacks (1 µm-spaced) were taken from brain sections of 4 mice/group/sex with a ZEISS Axiocam CCD monochrome camera and ZEN software (ZEISS Microscopy) at × 20 magnification. For the experiment 2, images (2048 × 2048 pixels, pixel size 0.33 µm) were obtained using a Leica Thunder Tissue 3D widefield fluorescence microscope (PRIMACEN platform, Rouen, France). Z-stacks (0.5 µm-spaced) were taken from 6 mice/group/sex using a DFC9000 GT VSC-12292 monochrome camera with Las X Navigator software (Leica Microsystems) at × 20 magnification. Identical illumination and exposure settings were applied for all images recorded. From the maximal intensity projection of these z-stacks, the GFAP- and IBA1 positive cells were counted manually and bilaterally by drawing region of interest (ROI) corresponding to the arcuate nucleus (ARC) of the hypothalamus using ImageJ software (National Institutes of Health, Bethesda, Maryland, US). The number of GFAP + and IBA1 + cells was calculated in each ROI (1 per hemisphere) /mm^2^ from 2–4 images per animal.

#### Confocal microscopy and 3D IMARIS analysis

For the 3D reconstruction of microglia and astrocytes, images (1024 × 1024 pixels, pixel size 0.23 µm) were obtained using a TCS SP8 DM6000B-CFS confocal microscope with a Leica DFC 365 Fx camera (× 63 oil-immersion lens, zoom 0.75). Z-stacks (0.3 µm steps) were taken in the ARC (1 image/hemisphere). All images were taken with the same confocal settings (pinhole, laser intensity, sequential mode). Raw files were then converted and analysed using IMARIS software (version 10.0.1, Oxford Instruments). First, the software was used to manually isolate cells using the cut function and to reconstruct the microglial surface using appropriate custom settings and to obtain volume object. Cells were included in the analysis when their soma was located within the central part of the z-stack and not cut by either the x or y plane (3–4 cells/hemisphere/animal). All morphological parameters were obtained after manually traced the IBA1 and GFAP staining using the Filament tracer mode. The volume as well as the number of total branch points and terminal points was measured for each cell. Branch Depth was defined as the number of branch points, or bifurcations, in the most complex path from the beginning point to a terminal point. Full Branch Level represented the highest value of Branching Level for the entire modeled cell. Filament Length corresponded to the sum of the lengths of all filaments. In addition, Sholl analysis was performed from the filament reconstruction mode.

### RNA extraction and real-time quantitative polymerase chain reaction (RT-qPCR)

The hypothalamus was homogenized in 500 µL of liquid TRIzol Reagent (Invitrogen). Then, the homogenized samples were incubated for 5 min at RT and 100 µL of liquid chloroform (Merck Millipore, Fontenay Sous Bois, France) were added, followed by centrifugation at 12000* g* (15 min, 4 °C). Following centrifugation, the aqueous phases containing the RNAs were transferred in new tubes and 250 µL of isopropanol was added to each tube. The samples were incubated for 10 min at RT, then centrifuged at 12000* g* at 4 °C. The precipitated RNA formed a white pellet on the bottom of the tubes. The supernatant was removed, and the pellets were washed 3 times with 75% ethanol, followed at each step by centrifugation (8000* g*; 3 min; 4 °C). The RNA pellets were air-dried before being resuspended in a volume of RNase-DNase free water (defined according to the size of the pellet obtained) for 1 h on ice.

Tubes contained RNA were incubated at 65 °C for 5 min. The total quantity and purity of the extracted RNA were determined by measuring the absorption at 260 and 280 nm using a Nanodrop 2000 spectrophotometer (Thermo Fisher Scientific).

For reverse transcription, each sample was diluted to obtain 1 μg of RNA in 8 μL of RNase-DNase free water. After adding 1 μL of 10X DNase Buffer (Invitrogen, Cat. No #AM8170G) and 1 μL of DNase (1 U/μL, RQ1 RNase-Free Dnase, Promega, Cat. No #M6101), the diluted samples were placed in a thermocycler (Eppendorf Mastercycler, Montesson, France) for 30 min at 37 °C. Then, samples were incubated for 10 min at 65 °C after the addition of 1 μL of DNase stop buffer to each tube. Then, 9 μL of the following mixture was added: 0.425 μL of RiboLock RNase Inhibitor (40 U/μL, Thermo Scientific, Cat. No # EO0381), 4 μL of 5X buffer, 1 μL of dNTP Set (Invitrogen, Cat. #10297018), 0.5 μL of Random Hexamer Primer (0.2 μg/μL Thermoscientific, Cat. No #SO142), 2 μL of DTT (0.1 M), 1 μL of M-MLV Reverse Transcriptase (200 U/μL, Invitrogen, Cat. #28025013), and 0.075 μL of DNase/RNase-Free Distilled Water (Invitrogen, Cat. No #10977015) per tube. Finally, the samples were incubated at 42 °C (50 min) followed by 5 min at 95 °C.

Real-time PCR was performed using the Thermocycler Bio-Rad C1000 Touch Real-Time Thermal Cycler CFX96 manager (Bio-Rad Laboratories, Marnes la Coquette, France). The cDNA products diluted (1:5) were used to carry out the PCR reactions. Moreover, a mix of cDNA products was also prepared to establish a standard curve. On a 96-well plate, 5 μL of SYBR Green mix (Bio-Rad, Cat. **#**1708880) and 0.9 μL of DNase/RNase-Free Distilled Water were added to each well in addition to 0.05 μL of reverse and 0.05 μL of foward primers (100 mM, Supplementary Table S2). Then, 4 μL of diluted cDNA products, standard samples or RNase-DNase free water (negative control) were added into this mixture. Each cycle consists of a denaturation phase at 95 °C, a hybridization phase whose temperature depends on each pair of primers (Tm) and a polymerization phase at 72 °C. mRNA levels were obtained from SQ (standard quantity) provided by Biorad software. The relative mRNA levels of genes of interest were normalized using two housekeeping genes (*β-actin* and *Gapdh*).

### Statistics

All the statistical analyses were performed with GraphPad Prism version 8.0.1 (GraphPad Software La Jolla, CA). The ROUT method was used to identify outliers with a Q coefficient equal to 1%. Data of experiment 1 (w14) were compared with 2-way ANOVA (HFD x sex) followed by Tukey post hoc tests except for body weight to initial weight ratio which was analysed using 3-way repeated measure ANOVA followed by Tukey post hoc tests comparing the difference between groups for each time point. For data of experiment 2 (d3/d7/d14/d28), values were compared with mixed-effect analysis (HFD x sex x time) followed by Tukey post hoc tests comparing the difference between groups for each time point. For the microscopy data, averaged values per animal were compared with 2-way ANOVA or mixed-effect analysis (HFD x sex x time or HFD x sex x distance) followed by Tukey post hoc tests. Details for each performed ANOVA are reported in Supplementary Table S3. All graphs are presented as mean ± standard error of the mean (SEM) with the following significance levels: *p < 0.05, **p < 0.01, ***p < 0.001, ****p < 0.0001.

## Results

### HFD consumption led to similar body weight gains in male and female mice after 14 weeks

**Fig. 1:**
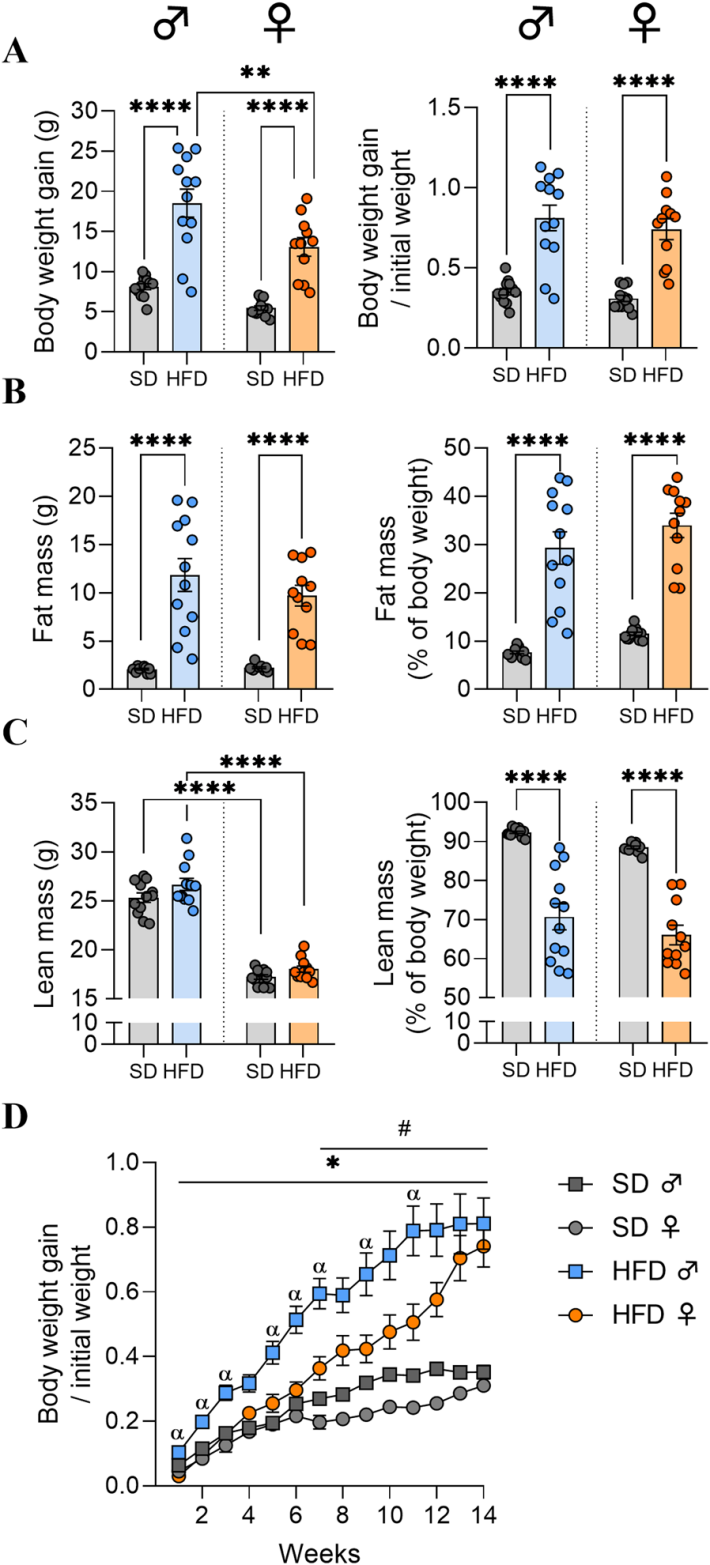
Monitoring of body weight and body composition after 14 weeks of high fat diet. **A** Body weight gain in grams (g) and body weight to initial weight ratio (expressed as percentage), **B** fat mass (g) and fat mass expressed as percentage of body weight, **C** Lean mass (g) and lean mass (% of body weight) measured by EchoMRI in C57Bl/6J male and female mice fed either a standard diet control (SD) or high fat diet (HFD) for 14 weeks (experiment 1, n = 12/group). **D** Body weight to initial weight ratio measured weekly for 14 weeks. Groups were compared with 2-way ANOVA followed by Tukey post hoc tests (*p < 0.05, **p < 0.01, ***p < 0.001, ****p < 0.0001) except in D panel where 3-way repeated measure ANOVA was used followed by Tukey post hoc tests (*p < 0.05 male SD *vs* HFD; #p < 0.05 female SD *vs* HFD; αp < 0.05 male HFD *vs* female HFD). Data are presented as mean ± standard error of the mean (SEM)

Male and female mice fed a high-fat diet (M- and F-HFD, respectively) gained more weight and exhibited more fat mass (in g and % of body weight) than control mice fed with a standard diet (SD; p < 0.0001, Fig. 1A, B) after 14 weeks (w14). More precisely, M-HFD and F-HFD gained in average 18.6 ± 1.7 g and 13.1 ± 1.2 g *vs* 8.1 ± 0.4 g and 5.5 ± 0.3 g for respective controls (M-SD and F-SD) and presented in average 11.9 ± 1.7 g (29.3 ± 3.4%) and 9.7 ± 1.1 g (34 ± 2.5%) *vs* 2.1 ± 0.1 g (7.6 ± 0.3%) and 2.2 ± 0.1 g (11.5 ± 0.3%) of fat mass (in g and % of body weight), respectively. In addition, a significant difference in body weight gain expressed in g between male and female HFD was observed (p < 0.01). There was no significant difference between the SD and HFD groups regarding lean mass (in g), however a significant difference was observed between male *vs* female animals fed with either SD or HFD (p < 0.0001, Fig. 1C). Nevertheless, the lean mass level (in % of body weight) was significantly decreased in both the M- and F-HFD groups (p < 0.0001, Fig. 1C). Weekly measured ratio of body weight gain to initial weight was significantly different between M-SD and M-HFD starting from w1 to w14, between F-SD and F-HFD from w7 to w14 and between F-HFD and M-HFD at w1-3, w5-7, w9 and w11 (Fig. 1D; p-values are detailed in Supplementary Table S3).

### HFD female mice exhibited a more pronounced satiety profile than male mice after 14 weeks

**Fig. 2:**
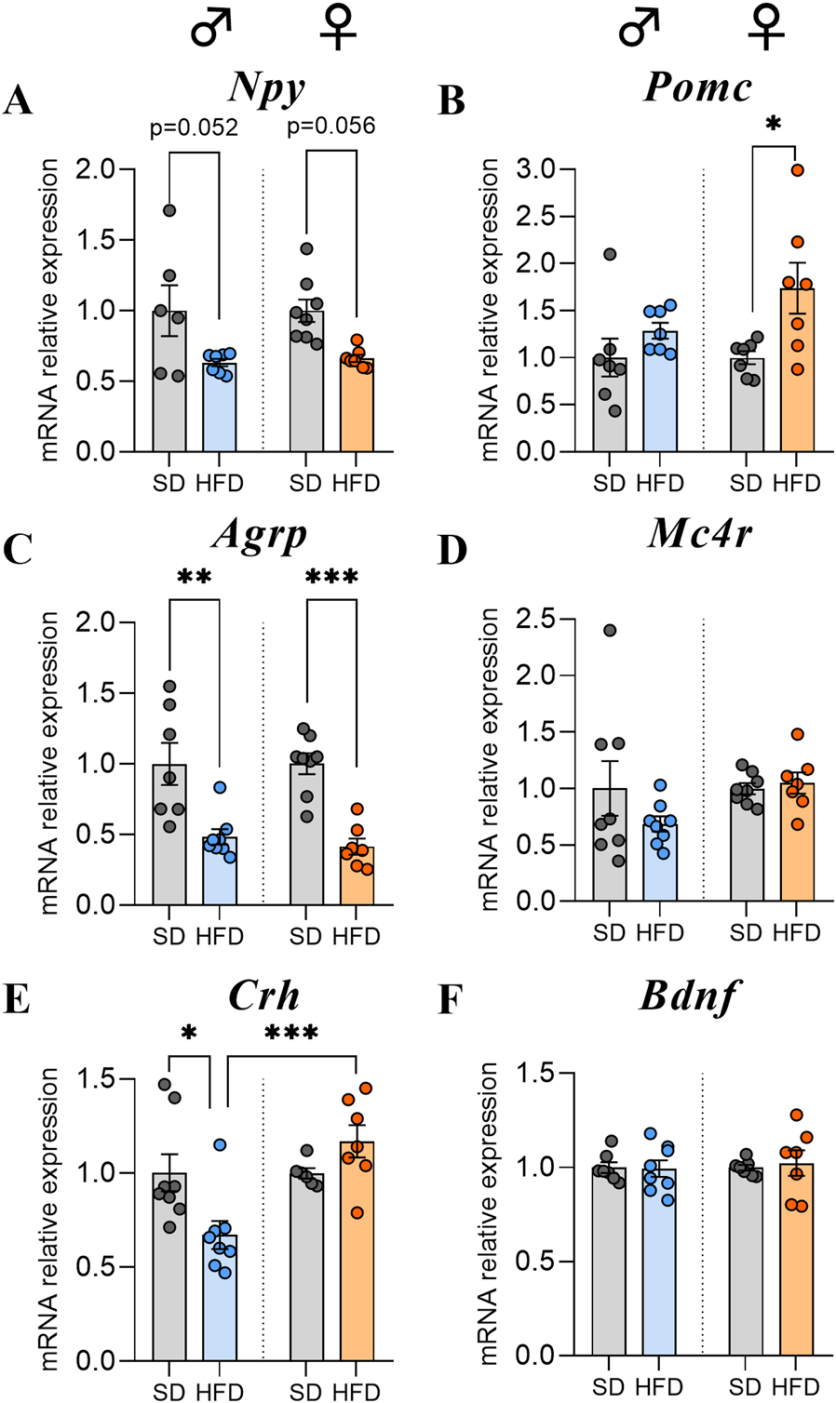
Effects of HFD for 14 weeks on hypothalamic mRNA expression of neuropeptides regulating food intake. **A**-**D** Relative quantification of mRNA transcript levels encoding orexigenic neuropeptides neuropeptide Y (*Npy*), agouti-related peptide (*Agrp*) and anorexigenic neuropeptides pro-opiomelanocortin (*Pomc*) and melanocortin-4 receptor (*Mc4r*) in hypothalamus of C57Bl/6J male and female mice fed for 14 weeks either SD or HFD (experiment 1, n = 8/group). **E**–**F** Relative quantification of mRNAs transcript levels encoding the corticotropin-releasing hormone (*Crh*) and brain-derived neurotrophic factor (*Bdnf*) in hypothalamus of C57Bl/6J male and female mice fed for 14 weeks either SD or HFD (experiment 1, n = 8/group). All mRNA levels were quantified relative to *Gapdh* and *β-actin* housekeeping gene expressions and compared with 2-way ANOVA followed by Tukey post hoc tests (*p < 0.05, **p < 0.01, ***p < 0.001). All graphs show the fold changes of mRNA levels for each HFD mouse compared to the mean of their respective control group. Data are presented as mean ± standard error of the mean (SEM)

The consumption of a hyperlipidic diet led to weight gain and greater fat storage in HFD-fed mice. Thus, we wanted to assess the effects of HFD feeding on signalling pathways involved in food intake and energy balance regulation. For that purpose, the levels of mRNA transcripts encoding orexigenic and anorexigenic neuropeptides were measured in the hypothalamus. The gene expression levels of neuropeptide Y (*Npy*) and agouti-related protein *(Agrp)* were downregulated both in M-HFD and in F-HFD (*Npy:* p = 0.052 and p = 0.056, respectively; *Agrp*: p < 0.01 and p < 0.001, respectively, Fig. 2A, C). Interestingly, the mRNA expression of the proopiomelanocortin *(Pomc)* gene was only upregulated in F-HFD (p < 0.05, Fig. 2B). Finally, the mRNA levels of the melanocortin-4 receptor *(Mc4r)* in HFD groups were not significantly different from those in the control groups in either sex after w14 (Fig. 2D).

### HFD male mice showed lower *Crh* gene expression levels after 14 weeks of HFD feeding

Corticotropin-releasing hormone (CRH) is known to coordinate the behavioural stress response [22] and is also involved in the control of energy homeostasis [41]. Thus, we were interested in the effects of HFD consumption on the gene expression regulation of this peptide. Interestingly, mRNA levels of *Crh* were decreased in M-HFD after w14 when compared to M-SD and F-HFD (p < 0.05 and p < 0.001, Fig. 2E). Because its expression could be regulated by nutritional state and by MC4R signalling [52], we also analysed the mRNA transcript encoding brain-derived neurotrophic factor (*Bdnf*). No significant difference was observed between the SD and HFD groups for either sex (Fig. 2F).

### HFD consumption did not induce a major hypothalamic inflammatory response in male or female mice after 14 weeks

**Fig. 3:**
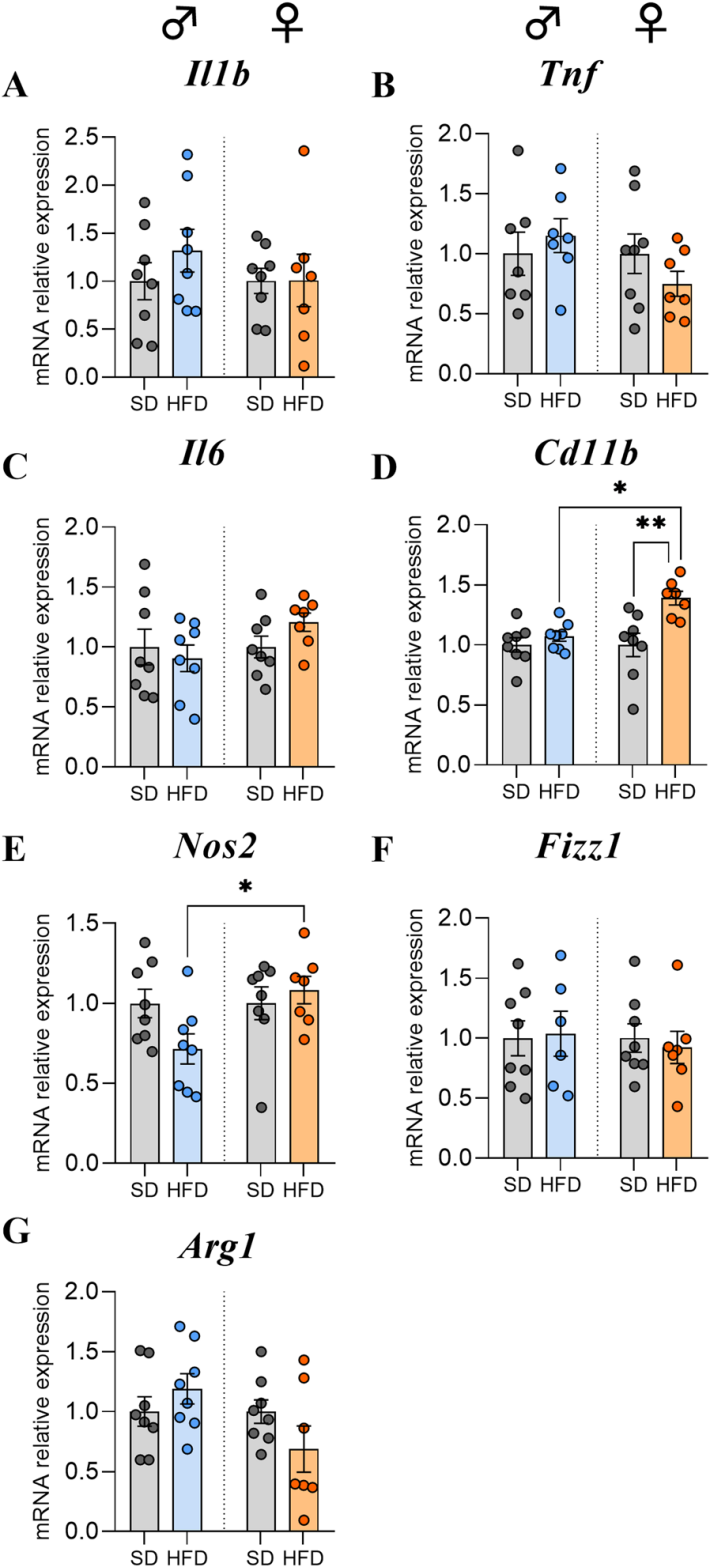
Effects of HFD feeding for 14 weeks on inflammatory marker expression levels in hypothalamus. **A**–**C** Relative quantification of mRNA transcript levels encoding proinflammatory cytokines with interleukin-1β (*Il1b*), tumor necrosis factor (*Tnf*), and interleukin-6 (*Il6*) in hypothalamus of C57Bl/6J male and female mice fed for 14 weeks either SD or HFD (experiment 1, n = 8/group). **D**–**G** Relative quantification of mRNA transcript levels encoding M1 polarization markers of macrophages (*Cd11b*, *Nos2*) and M2 polarization markers (*Fizz1, Arg1*) in hypothalamus of male and female C57Bl/6J mice fed for 14 weeks either SD or HFD (experiment 1, n = 8/group). All mRNA levels were quantified relative to *Gapdh* and *β-actin* housekeeping gene expressions and compared with 2-way ANOVA followed by Tukey post hoc tests (*p < 0.05, **p < 0.01). All graphs show the fold changes of mRNA levels for each HFD mouse compared to the mean of their respective control group. Data are presented as mean ± standard error of the mean (SEM)

After studying the expression profile of major hypothalamic neuropeptides, we next investigated the impact of a HFD on hypothalamic inflammatory markers. First, we analysed the mRNA expression levels encoding proinflammatory cytokines. Overall, no significant changes were found in the interleukin-1β (*Il1b*), interleukin-6 (*Il6*) or tumor necrosis factor (*Tnf*) levels in both sexes (Fig. 3A, B, C).

To continue, we were interested in gene expression profiling of macrophages by targeting pro- and anti-inflammatory phenotypes with the expression of M1 (*Cd11b, Nos2*) and M2 (*Fizz1*, *Arg1*) polarization markers. Interestingly, the mRNA level of the gene encoding *Cd11b* was only increased in F-HFD compared to F-SD and M-HFD (p < 0.01 and p < 0.05, Fig. 3D), while that of *Nos2* was only decreased in M-HFD compared to F-HFD group (p < 0.05, Fig. 3E). However, no significant changes in *Fizz1* or *Arg1* levels were found in male or female mice (Fig. 3F, G).

### Long-term HFD exposure induced the downregulation of Iba1 in female mice

**Fig. 4:**
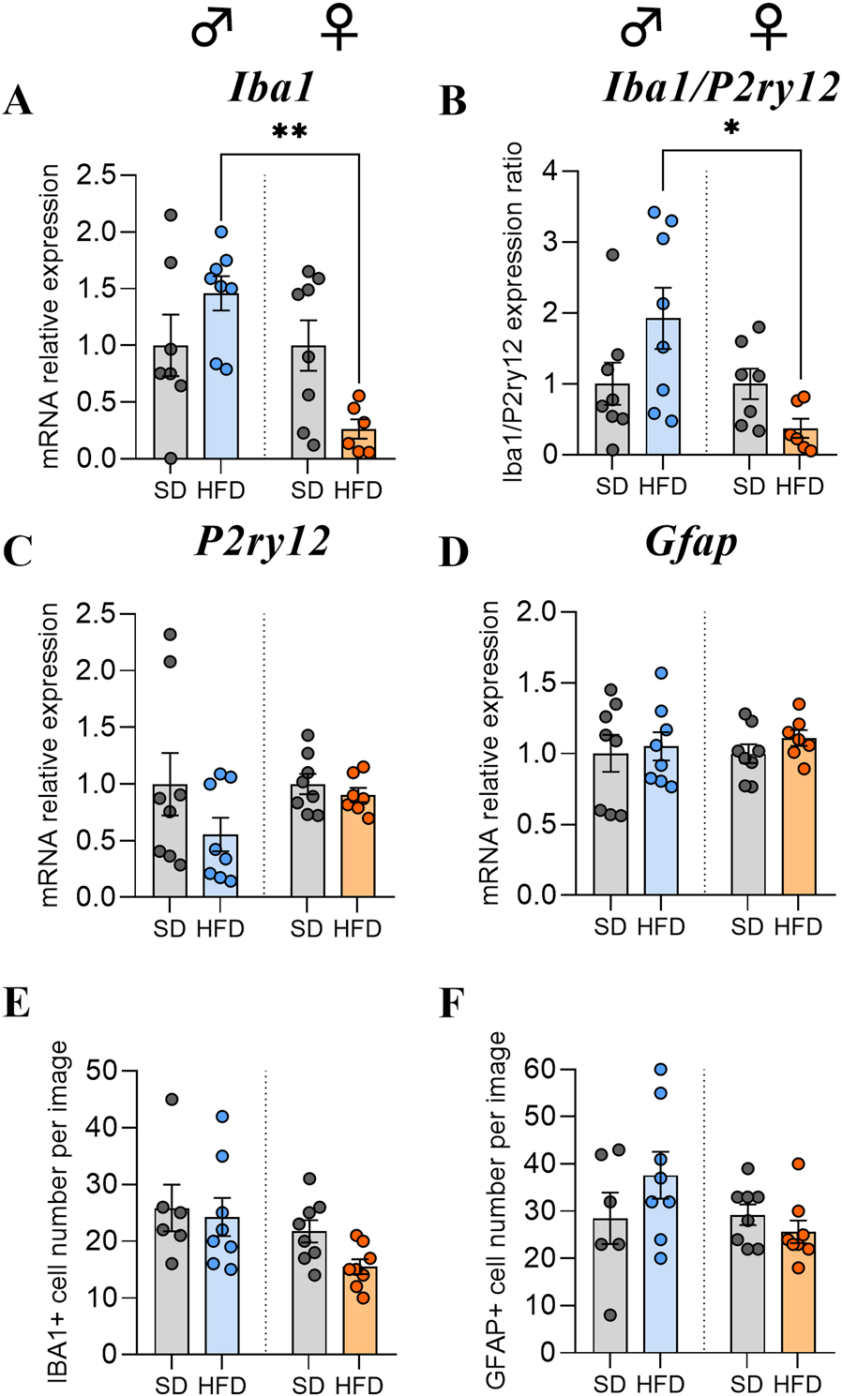
Effects of HFD feeding for 14 weeks on glial cell marker expression levels in hypothalamus. **A**–**D** Relative quantification of mRNA transcript levels encoding microglial markers with ionized calcium-binding adapter molecule 1 (*Iba1*), purinergic receptor P2Y12 (*P2ry12*) and astrocytic marker glial fibrillary acidic protein (*Gfap*) in hypothalamus of C57Bl/6J male and female mice fed for 14 weeks either SD or HFD (experiment 1, n = 8/group). All mRNA levels were quantified relative to *Gapdh* and β*-actin* housekeeping gene expressions and compared with 2-way ANOVA followed by Tukey post hoc tests (*p < 0.05, **p < 0.01). All graphs show the fold changes of mRNA levels for each HFD mouse compared to the mean of their respective control group. **E**–**F** Quantification of detection by immunofluorescence of IBA1 and GFAP proteins within the ARC from C57Bl/6J male and female mice fed for 14 weeks either SD or HFD (experiment 1, N = 2 images/animal with n = 4 mice/group). Immunopositive cells for IBA1 and GFAP were manually and bilaterally counted using Image J software in coronal sections of the ARC (20 µm, − 1.22 to 2.54 mm relative to Bregma). Data are presented as mean ± standard error of the mean (SEM). Averaged values per animal were compared with 2-way ANOVA followed by Tukey post hoc tests

According to the literature, a series of cellular and molecular events involving microglial cells and astrocytes, collectively termed "reactive gliosis", are observed in rodents fed with a HFD [25]. Therefore, we investigated the glial response by first performing RT‒qPCR of mRNA transcripts of genes classically associated with microgliosis and astrogliosis, such as the ionized calcium-binding adaptor molecule 1 (IBA1) protein, the purinergic receptor P2Y12 (P2RY12) and the glial fibrillary acidic protein (GFAP). HFD exposure for 14 weeks led to a decrease in the gene expression of the microglial marker *Iba1* in the hypothalamus as well as the *Iba1/P2ry12* ratio in F-HFD and an opposite trend in M-HFD in comparison to their respective SD groups, resulting in a significant difference between M-HFD and F-HFD (p < 0.01 and p < 0.05, respectively, Fig. 4A, B).

This quantification of multiple markers *via* RT‒qPCR was complemented by histological analysis with immunofluorescence staining of astrocytes and microglial cells in the ARC. Although no significant differences were observed regarding the number of IBA1 positive cells, 2-way ANOVA analysis revealed a significant sex-dependent effect (p(*sex*) < 0.05, Fig. 4E, Supplementary Fig. S1). Similar to the results of the *Gfap* gene expression analysis, the numbers of GFAP + cells in the ARC were equivalent between the SD-fed and HFD-fed mice (Fig. 4F, Supplementary Fig. S1).

These preliminary data did not seem to support the presence of hypothalamic inflammation but showed a sex-divergent response after long-term HFD exposure. Several studies have reported that hypothalamic inflammation occurs very early in response to a HFD before the onset of obesity [7, 47]. Therefore, we studied the hypothalamic response in a second cohort of male and female mice on a shorter timescale by monitoring the effects of 3, 7, 14 and 28 days of exposure to a HFD and performed the same analyses within the hypothalamus.

### HFD male mice gained more weight, body fat and more rapidly than HFD female mice during a short-term exposure

**Fig. 5:**
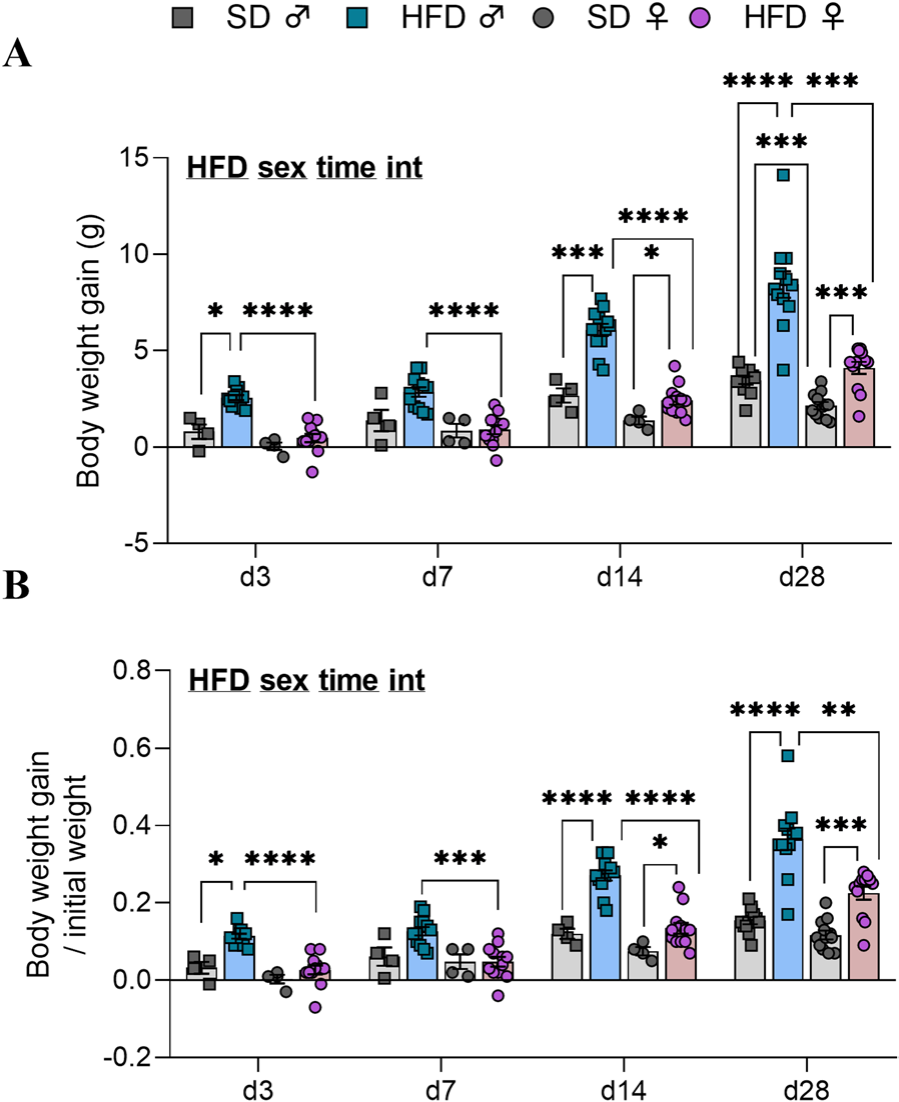
Body weight gain after 3, 7, 14 and 28 days of HFD feeding. **A** Body weight gain in grams (g) and **B** ratio of the body weight gain/initial weight in C57Bl/6J male (square individual values) and female (circle individual values) mice fed either SD (gray bars) or HFD (colored bars) feeding for up 28 days (experiment 2, n = 4 SD for d3, d7, d14; n = 12 SD for d28 and n = 12 HFD/time). Values were compared with mixed-effect analysis (sex x HFD x time) followed by Tukey post hoc tests (*p < 0.05, **p < 0.01, ***p < 0.001, ****p < 0.0001). In each graph, significant diet, sex, time and interaction effects are shown by bold and underlined text (HFD, sex, time, int). Data are presented as mean ± standard error of the mean (SEM)

**Fig. 6:**
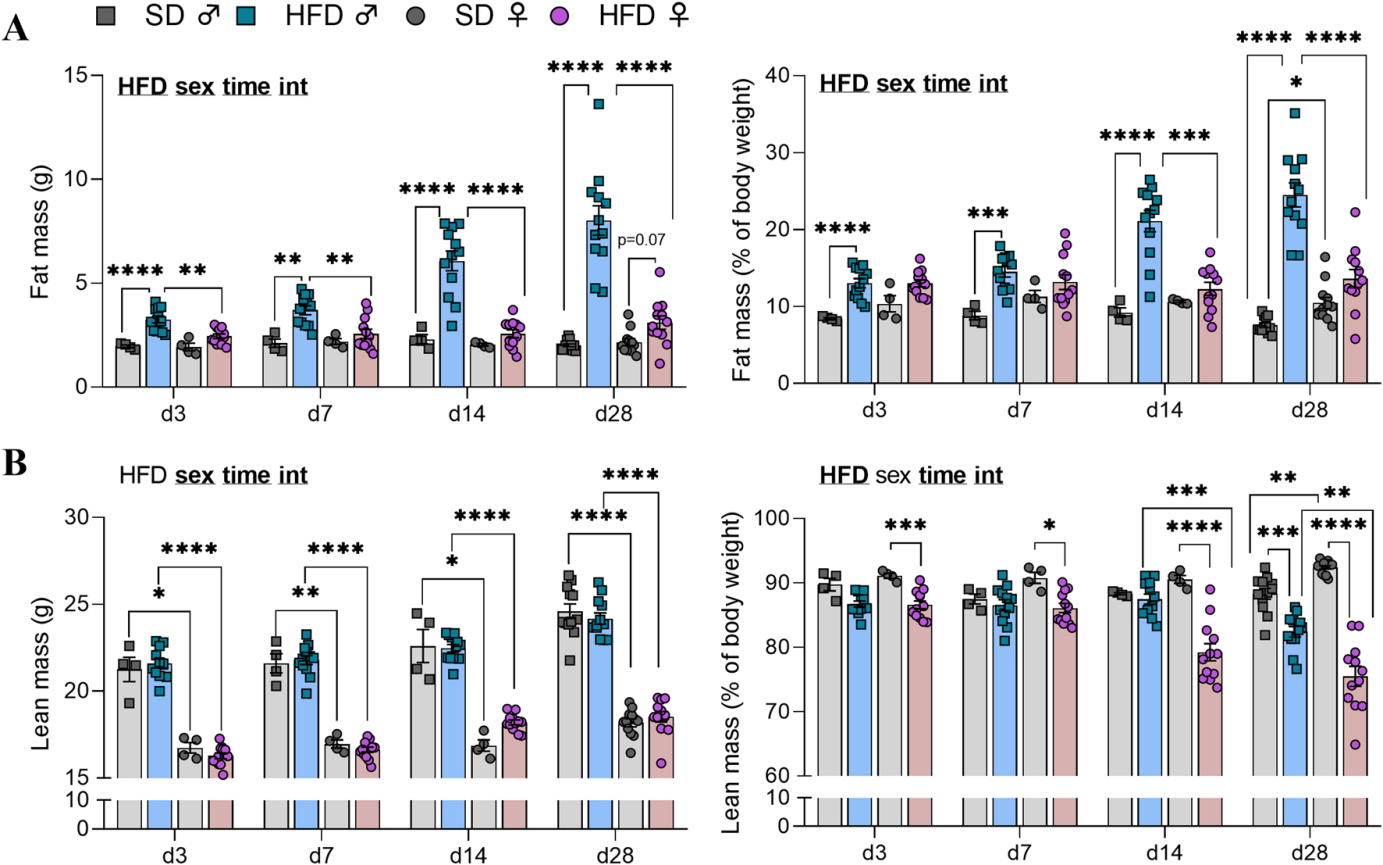
Body composition changes after 3, 7, 14 and 28 days of HFD feeding. **A** Fat mass expressed in grams and percentage of body weight and **B** lean mass (in g and % of body weight) measured by EchoMRI in C57Bl/6J male (square individual values) and female (circle individual values) mice fed either SD (gray bars) or HFD (colored bars) for up to 28 days (experiment 2, n = 4 SD for d3, d7, d14; n = 12 SD for d28 and n = 12 HFD/time). Values were compared with mixed-effect analysis (sex x HFD x time) followed by Tukey post hoc tests (*p < 0.05, **p < 0.01, ***p < 0.001, ****p < 0.0001). In each graph, significant diet, sex, time and interaction effects are shown by bold and underlined text (HFD, sex, time, int). Data are presented as mean ± standard error of the mean (SEM)

Overall, HFD consumption affected body weight gain and its ratio to initial weight (p(*HFD)* < 0.0001 for both measures*,* Fig. 5), fat mass (in g and %) (p(*HFD)* < 0.0001*,* Fig. 6A) and lean mass percentage (p(*HFD)* < 0.0001*,* Fig. 6B) in male and female mice. In addition, there was also a time exposure effect on body weight gain and its ratio to initial weight (p(*time)* < 0.0001, Fig. 5), on fat mass (in g and %) for both sexes (p(*time)* < 0.0001 and p(*time)* < 0.001, respectively Fig. 6A) and on lean mass (in g and %) for both sexes (p(*time)* < 0.0001, Fig. 6B).

More precisely, the body weight gain (in g) in male mice was significant compared to M-SD and F-HFD from d3 to d28 (M-SD *vs* M-HFD: p < 0.05 at d3, p < 0.001 at d14 and d28; M-HFD *vs* F-HFD: p < 0.0001 at d3-d14, p < 0.001 at d28, Fig. 5A). In contrast, female mice showed significant weight gain only from d14 to d28 of HFD (p < 0.05 and p < 0.001 *vs* F-SD, Fig. 5A). Interestingly, M-SD group differed from F-SD at d28 (p < 0.001). Similar variations were observed when body weight was normalised to initial weight (M-SD *vs* M-HFD: p < 0.05 at d3, d14 and d28; M-HFD *vs* F-HFD: p < 0.01 at d3, d7, d14 and d28; F-SD *vs* F-HFD: p < 0.05 at d14 and d28, Fig. 5B). On average, M-HFD gained 2.6 ± 0.1 g, 2.9 ± 0.2 g, 6 ± 0.3 g and 8.4 ± 0.7 g compared to 0.5 ± 0.2 g, 0.9 ± 0.2 g, 2.4 ± 0.2 g and 4.1 ± 0.3 g for F-HFD after d3, d7, d14 and d28, respectively (Fig. 5).

M-HFD-fed animals displayed an increase in fat mass at earlier time points than F-HFD-fed mice. Indeed, the fat mass (in g) was significantly greater in M-HFD mice starting from d3 to d28 compared to both M-SD and F-HFD (M-SD *vs* M-HFD: p < 0.01 at d3, d7, d14 and d28; M-HFD *vs* F-HFD: p < 0.01 at d3, d7, d14 and d28, Fig. 6A). Fat mass in F-HFD mice tended to be higher compared to F-SD only at d28 (p = 0.07 *vs* F-SD, Fig. 6A). When expressed as % of body weight, the fat mass increase was similar in M-HFD group when compared to M-SD, but less pronounced and appeared later (d14) when compared to F-HFD (M-SD *vs* M-HFD: p < 0.001 at d3, d7, d14 and d28; M-HFD *vs* F-HFD: p < 0.001 at d14 and d28, Fig. 6A). No increase between F-SD and F-HFD was seen, however fat mass percentage in F-SD group was higher than M-SD only at d28 (p < 0.05). Specifically, M-HFD exhibited greater fat mass than did F-HFD with in average fat masses of 3.3 ± 0.2 g (13 ± 0.6%), 3.7 ± 0.2 g (14.5 ± 0.7%), 6 ± 0.5 g (21.1 ± 1.4%) and 8 ± 0.7 g (24.5 ± 1.6%) compared to 2.4 ± 0.1 g (13 ± 0.5%), 2.6 ± 0.2 g (13.2 ± 0.9%), 2.6 ± 0.2 g (12.3 ± 0.8%), and 3.1 ± 0.3 g (13.6 ± 1.2%) for F-HFD at d3, d7, d14 and d28, respectively (Fig. 6A).

Absolute lean mass (g) was not significantly different between SD and HFD for both sexes (Fig. 6B), but a significant male *vs* female difference was seen for each time point (M-SD *vs* F-SD: p < 0.05; M-HFD *vs* F-HFD: p < 0.0001, Fig. 6B). On the other hand, the proportion of lean mass (in %) decreased for F-HFD from d3 to d28 and only at d28 for M-HFD (F-SD *vs* F-HFD: at least p < 0.05 at d3, d7, d14 and d28; M-SD *vs* M-HFD: p < 0.001, Fig. 6B). Sex-dependent differences were only observed at d28, where female SD mice had a higher relative lean mass than their male counterpart and where F-HFD mice had a lower percentage than M-HFD (p < 0.01 for both).

### Short-term HFD exposure led to specific modulation of neuropeptide gene expression involved in energy balance regulation in a sex- and time-dependent manner between male and female mice

**Fig. 7:**
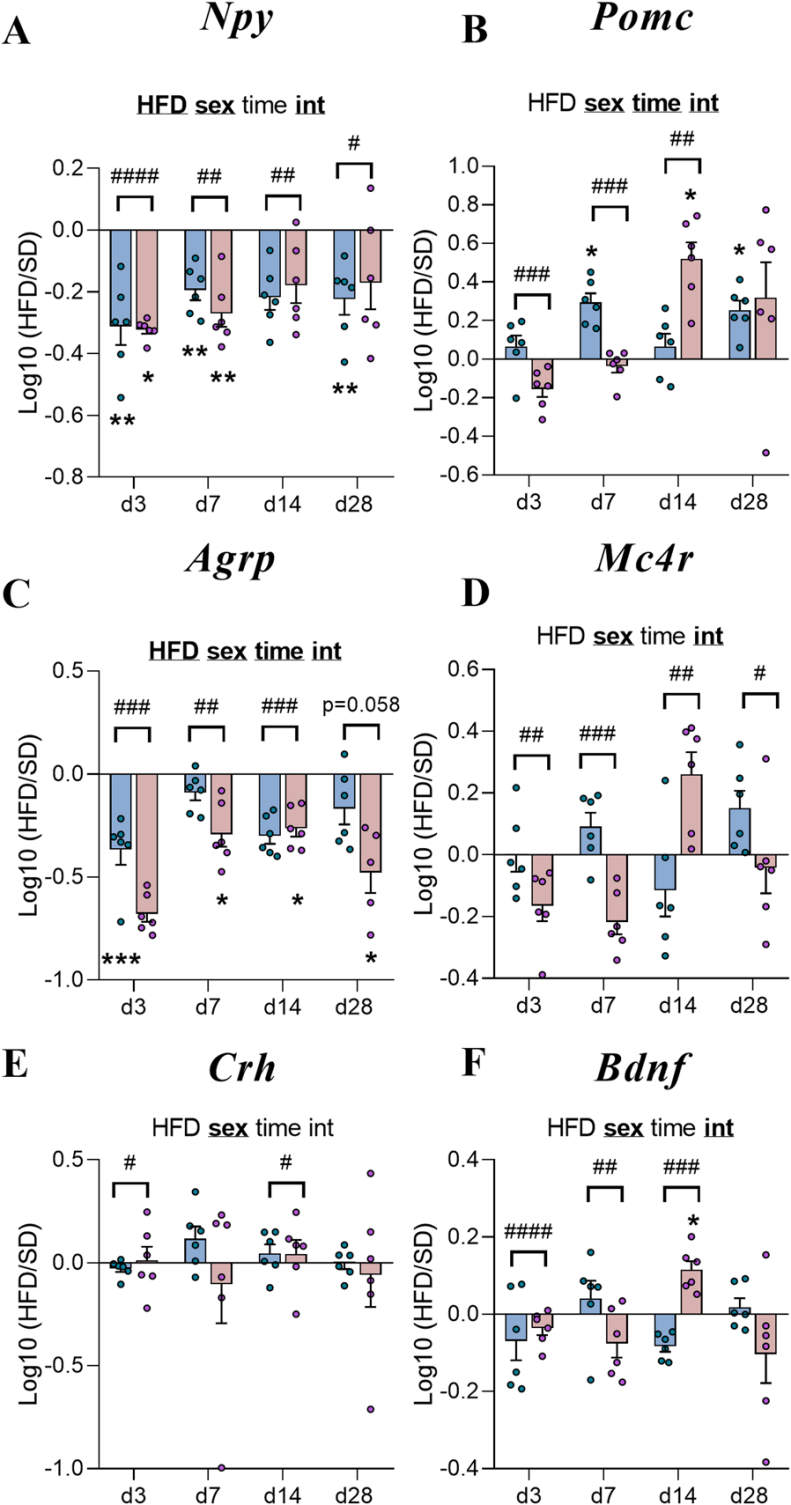
Time course response to HFD of hypothalamic neuropeptide gene expressions involved in food intake regulation. **A**-**F** Graphs show the fold changes of mRNA transcript levels encoding orexigenic neuropeptides with neuropeptide Y (*Npy*), agouti-related peptide (*Agrp*), anorexigenic neuropeptides with pro-opiomelanocortin (*Pomc*), the melanocortin-4 receptor (*Mc4r*) as well as the corticotropin-releasing hormone (*Crh*) and the brain-derived neurotrophic factor (*Bdnf*) of HFD male and HFD female mice (blue and pink bars, respectively) compared to the mean of their respective control group (Log10 (HFD/SD)) after 3, 7, 14 and 28 days (d3, d7, d14, d28, experiment 2, n = 6/group). All mRNA species were quantified relative to *Gapdh* and *β-actin* housekeeping gene expression. Raw data were first compared with mixed-effect analysis (sex x HFD x time) followed by Tukey post hoc tests (*p < 0.05, **p < 0.01, ***p < 0.001 SD *vs* HFD; #p < 0.05, ##p < 0.01, ###p < 0.001, ####p < 0.0001 male HFD *vs* female HFD). In each graph, significant diet, sex, time and interaction effects are shown by bold and underlined text (HFD, sex, time, int). Data are presented as mean ± standard error of the mean (SEM)

Consistently with the first cohort (experiment 1), the expression of mRNAs encoding NPY and AgRP were also decreased in male and female mice after short-term HFD consumption (p(*HFD*) < 0.0001, p(*time*) < 0.01) and differed between two sexes (p(*sex*) < 0.0001, p(*int*) < 0.001, Fig. 7A, C**)**. Indeed, mRNA levels encoding NPY were significantly decreased at d3, d7 and d28 for M-HFD (p < 0.01 *vs* M-SD) and at d3 and d7 for F-HFD (p < 0.05 *vs* F-SD) with a significant difference between male and female mice for each time (p < 0.05, Fig. 7A). In the same way, *Agrp* levels were significantly decreased from d7 up to d28 for F-HFD (p < 0.05 *vs* F-SD) in contrast to only at d3 for M-HFD (p < 0.001 *vs* M-SD, Fig. 7C) in a sex-dependent manner from d3 to d14 (p < 0.01) and a strong trend at d28 (p = 0.058, Fig. 7C). The significant upregulation of *Pomc* gene expression found in F-HFD mice at w14 (Fig. 2B) was also observed but only at d14 (p < 0.05 *vs* F*-*SD*,* Fig. 7B) and also in M-HFD, specifically at d7 and d28 (p < 0.05 *vs* M*-*SD). Additionally, significant sex and time effects (p(*sex*) < 0.0001, p(*time*) < 0.01, p(*int*) < 0.01) were observed from d3 to d14 (p < 0.01, Fig. 7B). While HFD consumption did not affect the mRNA levels of the genes encoding MC4R, CRH and BDNF in both sexes compared to what was observed in M-HFD (especially for the *Crh* levels after w14; Fig. 2E), a significant sex effect was reported for these three factors (p(*sex*) < 0.001; Fig. 7D, E, F).

### Short-term HFD exposure was associated with a sex-dependent expression of inflammatory markers in mice

**Fig. 8:**
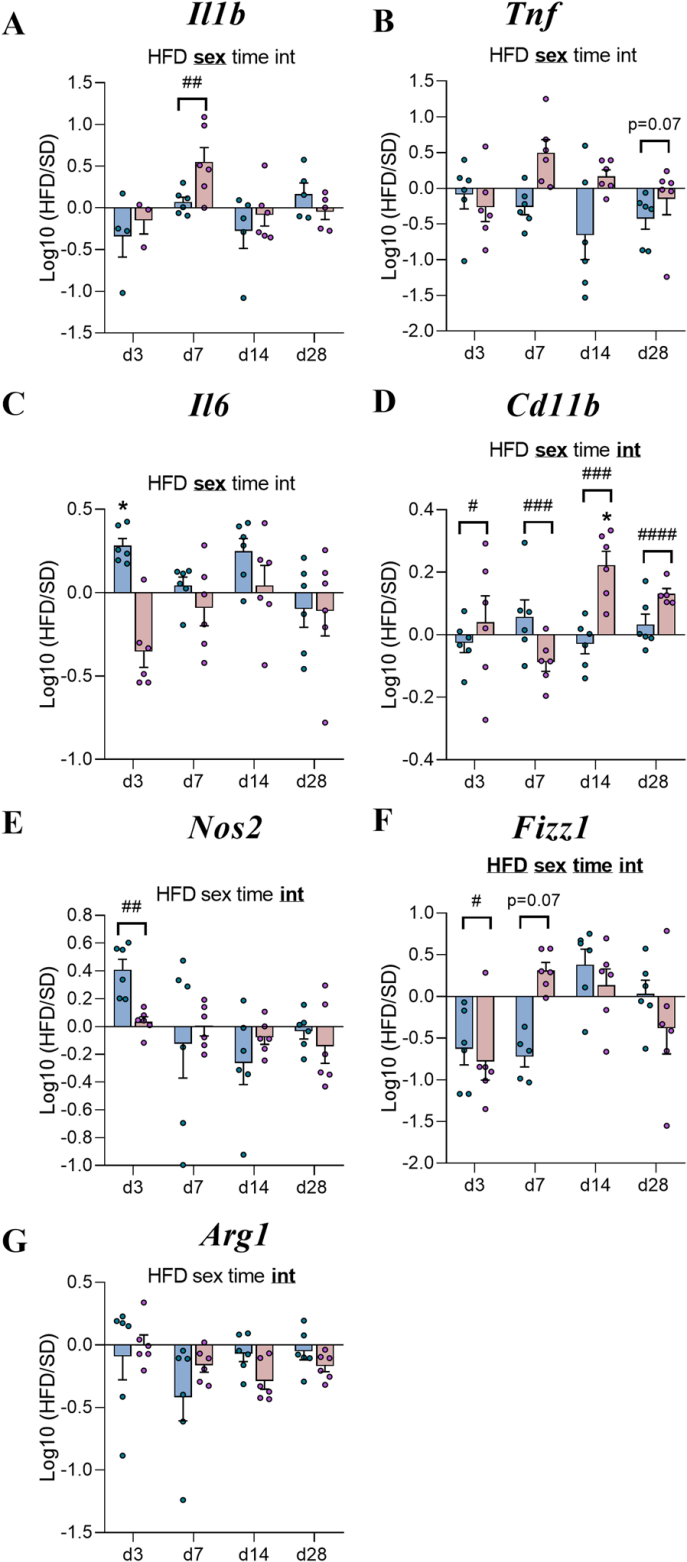
Time course response to HFD of hypothalamic inflammatory markers. **A**-**G** Graphs show the fold changes of mRNA transcript levels encoding proinflammatory cytokines with interleukin-1β (*Il1b*), tumor necrosis factor (*Tnf*), interleukin-6 (*Il6*), M1 polarization markers of macrophages (*Cd11b*, *Nos2*) and M2 polarization markers (*Fizz1*, *Arg1*) of HFD male and HFD female mice (blue and pink bars, respectively) compared to the mean of their respective control group (Log10 (HFD/SD)) after 3, 7, 14 and 28 days (d3, d7, d14, d28, experiment 2, n = 6/group). All mRNA species were quantified relative to *Gapdh* and *β-actin* housekeeping gene expression. Raw data were first compared with mixed-effect analysis (sex x HFD x time) followed by Tukey post hoc tests (*p < 0.05 SD *vs* HFD; #*p* < 0.05, ##*p* < 0.01, ###*p* < 0.001, ####*p* < 0.0001 male HFD *vs* female HFD). In each graph, significant diet, sex, time and interaction effects are shown by bold and underlined text (HFD, sex, time, int). Data are presented as mean ± standard error of the mean (SEM)

As observed in the first experiment, mRNA levels of proinflammatory cytokines in HFD-fed mice were not significantly different from those in SD mice except to a transient increase of *Il6* levels in M-HFD at d3 (p < 0.05 *vs* M-SD, Fig. 8C). Nevertheless, it was reported a main sex effect especially at d7 for *Il1b* levels (p < 0.01, Fig. 8A) and d28 for *Tnf *levels (p = 0.07, Fig. 8B). Interestingly, the upregulation of *Cd11b *observed at w14 in F-HFD compared to F-SD (Fig. 3D) also occurred at d14 (p < 0.05, Fig. 8D) and the mRNA expression levels were significantly different compared to male mice from d3 (p < 0.05 Fig. 8D). Moreover, there was also significant sex differences at d3 for *Nos2* as well as *Fizz1* levels *(*p < 0.05, Fig. 8E, F).

### Short-term HFD exposure did not induce relevant changes in genes related to glial markers in mice but differed by gender

**Fig. 9:**
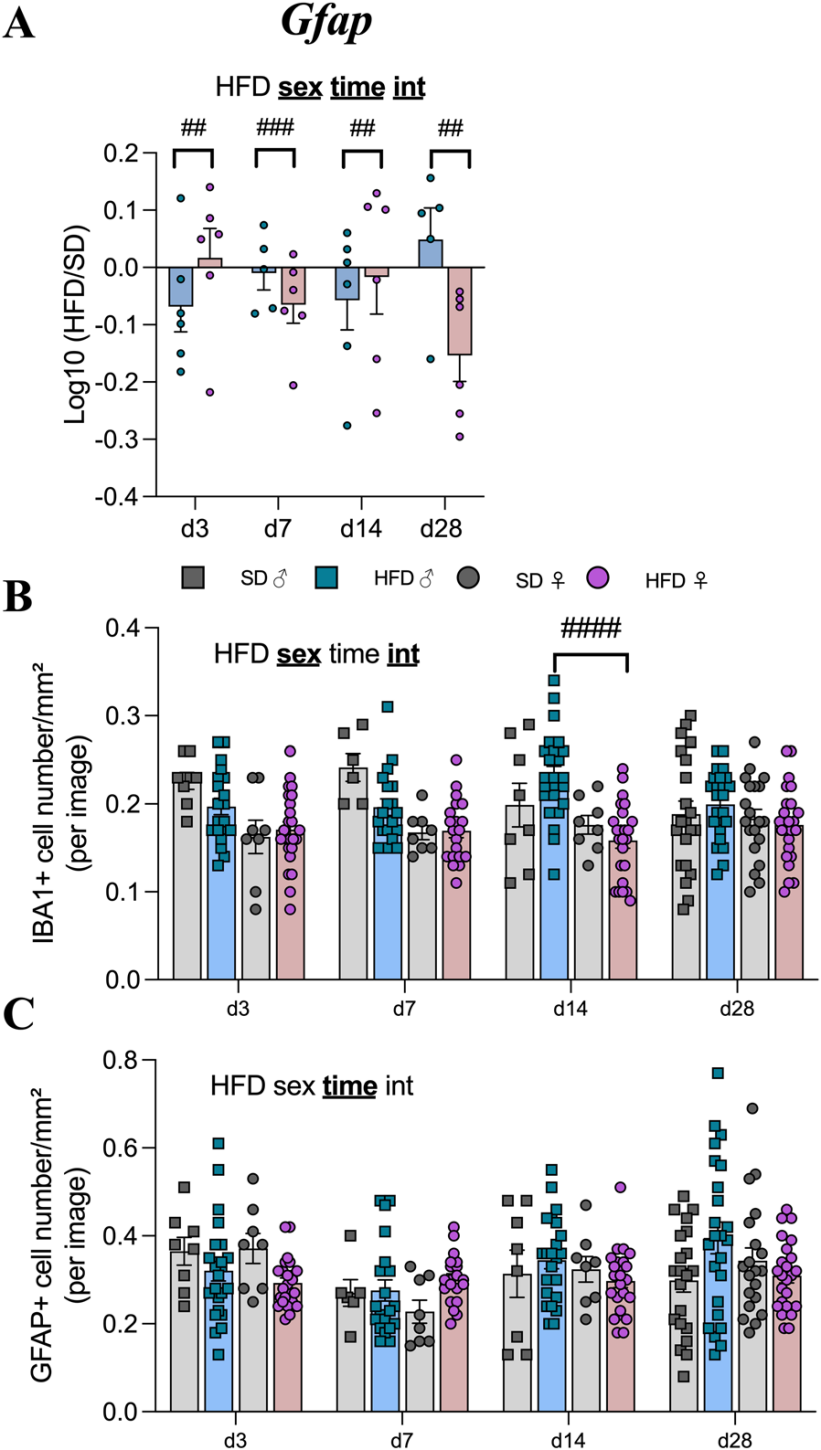
Time course response to HFD of glial marker levels in hypothalamus. **A** Graph show the fold changes of mRNA transcript levels encoding astrocytic marker glial fibrillary acidic protein (*Gfap*) of HFD male and HFD female mice (blue and pink bars, respectively) compared to the mean of their respective control group (Log10 (HFD/SD)) after 3, 7, 14 and 28 days (d3, d7, d14, d28, experiment 2, n = 6/group). The mRNA species were quantified relative to *Gapdh* and *β-actin* housekeeping gene expression. **B**, **C** Quantification of detection by immunofluorescence of IBA1 and GFAP proteins within the ARC from C57Bl/6J male (square individual values) and female (circle individual values) mice fed for 3,7,14 and 28 days either SD (gray bars) or HFD (colored bars; N = 4 images/animal with n = 6 mice/group). Immunopositive cells for IBA1 and GFAP were manually and bilaterally counted using Image J software in coronal sections of the ARC (20 µm, − 1.22 to 2.54 mm relative to Bregma). Raw data (**A**) or average number of cells per animal (**B** and **C**) were first compared with mixed-effect analysis (sex x HFD x time) followed by Tukey post hoc tests (##p < 0.01, ###p < 0.001, ####p < 0.0001). In each graph, significant diet, sex, time and interaction effects are shown by bold and underlined text (HFD, sex, time, int). Data are presented as mean ± standard error of the mean (SEM)

In contrast to previous studies [47] and although a decrease in *Iba1* expression occurred in F-HFD at w14 (Fig. 4A), we did not observe significant effects of HFD on *Iba1* and *P2ry12* gene expressions. However, mRNA encoding Iba1, P2ry12 as well as Iba1/P2ry12 ratio level significantly differed between male and female mice (p*(sex)* < 0.0001, see Supplementary Table S3). Moreover, time-dependent downregulation of *Gfap* gene expression was observed in HFD mice (p(*time)* < 0.001, Fig. 9A) which differed according the sex from d3 to d28 (p < 0.01, Fig. 9A). Immunofluorescence analyses revealed sex effect on the number of microglial cells (p*(sex)* < 0.0001; p(*time x HFD x sex)* < 0.05) and time effect on the number of astrocytes in the ARC (p(*time*) < 0.01, Fig. 9B, C). More precisely, a downward trend in the number of IBA1 + cells in female mice was specifically significant in F-HFD compared to M-HFD mice at d14 (p < 0.0001, Fig. 9B).

### Signs of structural remodeling of microglia and astrocytes differed between male and female mice after 28 days and 14 weeks of HFD feeding

**Fig. 10:**
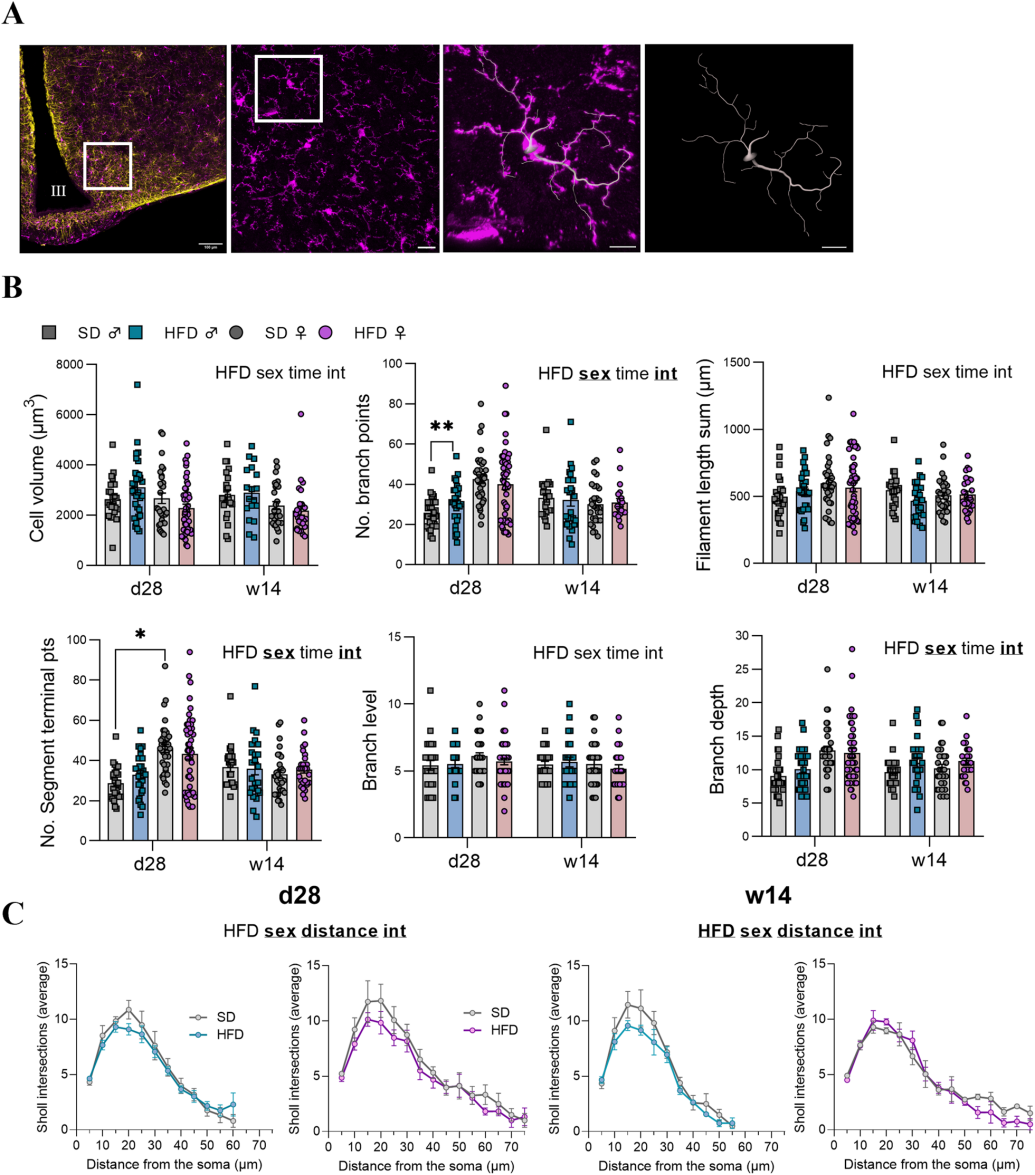
Morphometric analysis of microglia after 28 days and 14 weeks of HFD within the ARC. **A** First panel: representative image of a 3D mosaic (512 × 512 pixels, voxel size 0.459 µm) from confocal microscope showing IBA1, GFAP and merge stained microglia (purple) and astrocytes (yellow) within the ARC. Scale bar: 100 µm, III: third ventricle. Second panel: representative image showing IBA1 staining obtained after maximal projection Z-stacks (1024 × 1024 pixels, 234.55 × 234.55 µm). Scale bar: 20 µm. Third panel: focus of the white square drawn in the second panel which highlights an example of the filament tracing performed in isolated microglia. Scale bar: 8 µm. **B** Graphs show the values of each individual microglial cell for cell volume, Filament No. segment branch points/terminal points, filament full branch depth/level, and filament length sum for male (square individual values) and female (circle individual values) mice fed for 28 days (n = 3–4 cells/hemi-ARC from n = 6 mice/group) or 14 weeks (n = 3–4 cells/hemi-ARC from n = 4 mice/group) either SD (gray bars) or HFD (blue and pink bars for male and female mice, respectively). Averaged values per animal were first compared with mixed-effect analysis (sex x HFD x time) followed by Tukey post hoc tests (*p < 0.05, **p < 0.01). In each graph, significant diet, sex, time and interaction effects are shown by bold and underlined text (HFD, sex, time, int). Data are presented as mean ± standard error of the mean (SEM). **C** Graphs show the mean distribution of the number of Sholl intersections as a function of the distance from the microglial soma for SD mice (grey curves) and HFD mice (blue and pink curves for male and female mice, respectively) at d28 (n = 3–4 cells/hemi-ARC from 6 mice/group) and at 14 weeks (n = 3–4 cells/hemi-ARC from 4 mice/group). Averaged values per animal were first compared with mixed-effect analysis (sex x HFD x distance) followed by Tukey post hoc tests (*p < 0.05). In each graph, significant diet, sex, time,distance and interaction effects are shown by bold and underlined text (HFD, sex, distance, int). Data are presented as mean ± standard error of the mean (SEM)

**Fig. 11:**
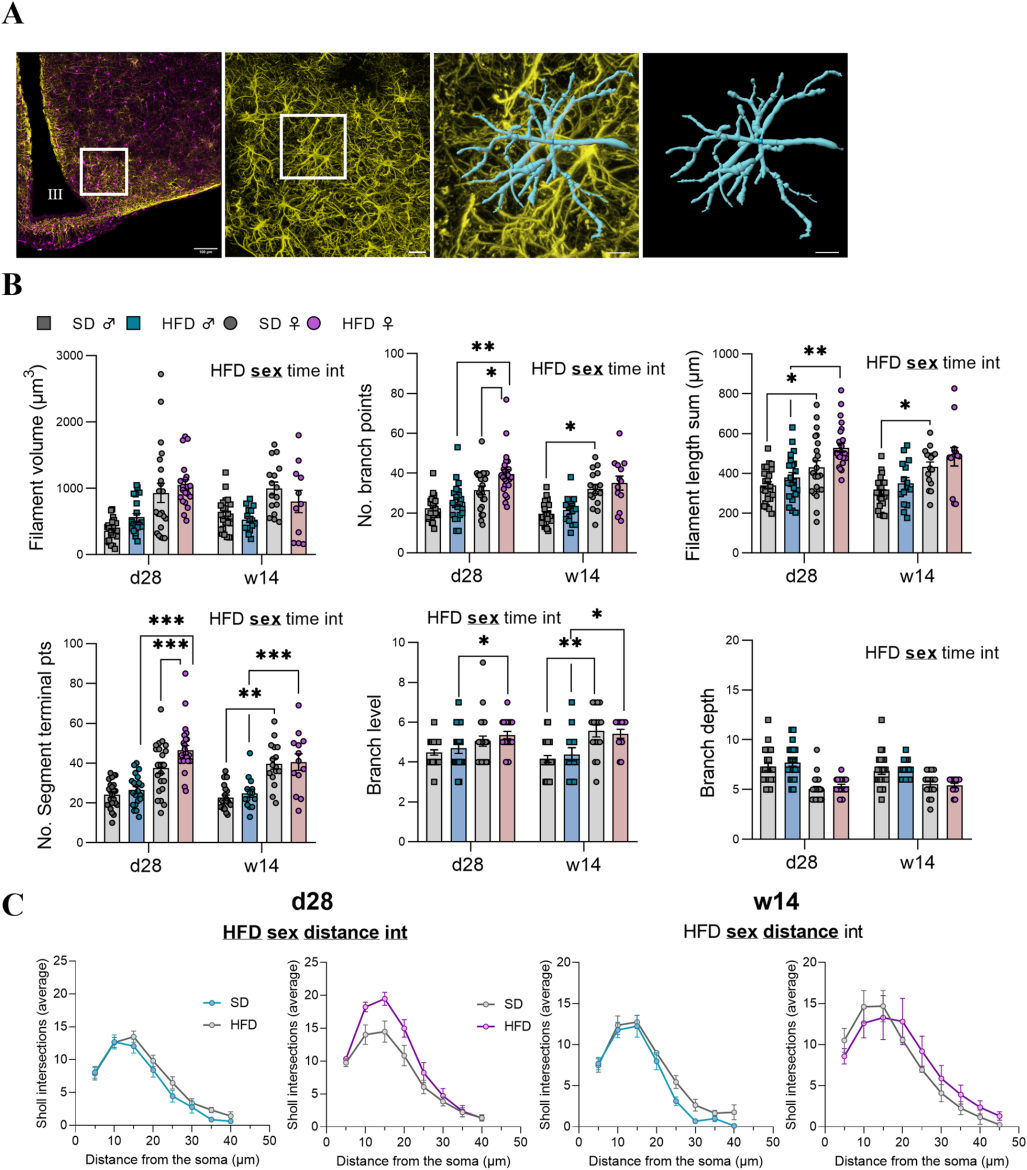
Morphometric analysis of astrocytes after 28 days and 14 weeks of HFD within the ARC. **A** First panel: representative image of a 3D mosaic (512 × 512 pixels, voxel size 0.459 µm) from confocal microscope showing IBA1, GFAP and merge stained microglia (purple) and astrocytes (yellow) within the ARC. Scale bar: 100 µm, III: third ventricle. Second panel: representative image showing GFAP staining obtained after maximal projection Z-stacks (1024 × 1024 pixels, 234.55 × 234.55 µm). Scale bar: 20 µm. Third panel: focus of the white square drawn in the second panel highlights an example of the surface reconstruction of an isolated astrocyte. Scale bar: 8 µm. **B** Graphs show the values of each individual astrocytes for Filament volume sum, Filament No. segment branch points/terminal points, filament full branch depth/level, and filament length sum for male (square individual values) and female (circle individual values) mice fed for 28 days(n = 3–4 cells/hemi-ARC from n = 6 mice/group) or 14 weeks (n = 3–4 cells/hemi-ARC from n = 4 mice/group) either SD (gray bars) or HFD (blue and pink bars for male and female mice, respectively). Averaged values per animal were first compared with mixed-effect analysis (sex x HFD x time) followed by Tukey post hoc tests (*p < 0.05, **p < 0.01, p*** < 0.001). In each graph, significant diet, sex, time and interaction effects are shown by bold and underlined text (HFD, sex, time, int). Data are presented as mean ± standard error of the mean (SEM). **C** Graphs show the mean distribution of the number of Sholl intersections as a function of the distance from the astrocyte soma for SD mice (grey curves) and HFD mice (blue and pink curves for male and female mice, respectively) at d28 (n = 3–4 cells/hemi-ARC from 6 mice/group) and at w14 (n = 3–4 cells/hemi-ARC from 4 mice/group). Averaged values per animal were first compared with mixed-effect analysis (sex x HFD x distance) followed by Tukey post hoc tests (*p < 0.05). In each graph, significant diet, sex, time and interaction effects are shown by bold and underlined text (HFD, sex, distance, int). Data are presented as mean ± standard error of the mean (SEM)

To further assess the glial response, we implemented three-dimensional morphometric analyses of individual microglial cells and astrocytes in the ARC at d28 and w14 after HFD feeding. Using IMARIS software, we assessed various features, including cell volume, glial branch parameters (filament length, filament full branch depth/level, and filament number segment branch/terminal points) and Sholl intersections. Interestingly, we firstly reported remodelling of microglia in male mice and overall, a sex and interaction (time x sex) effects were observed in segment branch points, terminal points and branch depth (see Supplementary Table S3). More specifically, M-HFD and F-SD mice at d28 displayed microglia with more segment branch points and terminal points compared to M-SD (p < 0.05; Fig. 10B). Second, we found significant morphometric changes in astrocytes, such as an increase in the number of branch points in F-HFD compared to F-SD and M-HFD at d28 (p < 0.05 and p < 0.01, respectively, Fig. 11B) and a significant increase in F-SD compared to M-SD at w14 (p < 0.05). Total filament length was shorter in M-SD in comparison to F-SD at both times (p < 0.05 for both) and in M-HFD *vs* F-HFD at d28 (p < 0.01). The number of terminal points was increased in F-HFD vs F-SD at d28 (p < 0.001) and vs M-HFD at d28 and w14 (p < 0.001 for both time points). In addition, at w14, F-SD was higher than M-SD for this measure (p < 0.01). Last, higher branch level in female was observed for the two time points in HFD groups and at w14 for SD groups (M-SD *vs* F-SD: p < 0.01 at w14; M-HFD *vs* F-HFD: p < 0.05 at d28 and w14; Fig. 11B). For each criteria, a sex effect was observed following mixed-effect analysis (see Supplementary Table S3 for details).

Proinflammatory microglia have been demonstrated to release inflammatory cytokines and especially undergo deramification characterized by the retraction of their processes with loss of complexity [5]. Thus, we performed a Sholl analysis of individual 3D-reconstructed cells. To this end, spheres with a radius of 5 µm were superimposed starting at the center of the soma, and the number of process intersections that each sphere encountered was measured with IMARIS software. Overall, a diet effect was seen in w14 for microglia and in d28 for astrocytes but no change in HFD compared to SD (for both sexes) were revealed by post-hoc analysis for microglial cells and astrocytes. Nevertheless, mixed effect analysis revealed a sex effect and a distance effect for microglia and astrocytes at both time points (see Supplementary Table S3 for details on p-values and interaction effect; Figs. 10C and 11C).

## Discussion

In these two experiments, we investigated the impact of obesity on the hypothalamic response by using a DIO model. Indeed, DIO models with HFD consumption are commonly used to induce obesity in rodents [10]. Starting at 7 weeks of age, the first cohort of C57Bl/6J male and female mice received either a SD or a HFD with 60% kcal from fat for 14 weeks. First, the monitoring of body weight and body composition allowed us to confirm the robustness of our model. Indeed, compared to control mice, both M- and F-HFD-fed mice gained significantly more weight and exhibited significant changes in body composition with a greater increase in fat mass.

Regarding sex differences in the response to HFD, we first noted that F-HFD mice exhibited lower absolute weight gain than male mice at the end of 14 weeks. However, when we calculated the final body weight gain to initial weight ratio, we noticed that the normalized increase was similar between male and female mice, suggesting that the HFD had similar effect on male *versus* female mice, at least for the weight gain criteria. Nevertheless, the weight gain to initial weight ratio over the course of the 14 weeks exposure to HFD was significantly different between male and female animals. Indeed, the increase in weight gain appeared earlier in M-HFD than F-HFD mice. This observation was already reported by other studies where males respond faster to HFD than do females mice [53]. Ovariectomy in female mice eliminates protection against weight gain, and results in a profile similar to that of male mice [20]. Estrogen levels may contribute to sex differences in response to DIO. Indeed, estrogens protect against increased adiposity and obesity through satietogenic effects. Estradiol suppresses feeding by enhancing the potency of other anorectic signals, such as cholecystokinin, leptin, BDNF, and by decreasing the potency of orexigenic signals such as melanin-concentrating hormone and ghrelin. Rodent studies also identified that activation of the estrogen receptors in the ventral medial nucleus of the hypothalamus results in increased energy expenditure [33].

Besides the impact of these factors, differences in susceptibility to DIO could be related to diet composition. Indeed, given the different composition of the HFD when compared to control SD, we wanted to assess the impact of a HFD at this time point on regulatory molecules of energy homeostasis and food intake. We hypothesized HFD could present strong satietogenic potential due to its high caloric density (5.24 kcal/g *vs* 3.34 kcal/g for SD). This was supported by weekly food consumption per cage from week 1 to week 12. Indeed, we reported a significant main effect of HFD and sex: a lower food intake expressed in g in HFD mice compared to SD but a higher caloric intake, which was more pronounced in males compared to females (Supplementary Fig. S2). It has been documented that a HFD induces neuronal adaptations that affect central neuropeptides involved in mediating satiety [19]. For instance, studies have reported that dietary fats activate orexigenic neurons coexpressing neuropeptide Y (NPY)/Agouti-related protein (AgRP) while also attenuating anorexigenic neurons coexpressing proopiomelanocortin (POMC)/cocaine- and amphetamine-related transcript (CART), which contributes to hyperphagia and eventually leads to obesity [19]. Interestingly, we found opposite results in HFD-fed mice, with a downregulation of mRNA transcripts encoding orexigenic neuropeptides and an upregulation of those encoding POMC but only in the F-HFD group. Furthermore, the increase in fat mass in HFD mice was also accompanied by glucose intolerance and higher insulin and leptin plasma concentrations compared to SD mice [26]. Our findings regarding food intake data support a satietogenic effect of a HFD and could reflect adaptative feedback mechanisms following HFD or suggest a potential leptin dependant effect in our model. Indeed, leptin directly influences the secretion patterns of NPY and AgRP peptides as well as POMC and CART peptides, either by inhibiting or activating neurons after binding to leptin receptors in the arcuate nucleus of the hypothalamus [19]. In our first experiment, although there was no main sex effect, only female mice showed increased expression levels of *Pomc* mRNA in HFD conditions. This was not observed in male mice. Our results may support the principle that female rodents are more sensitive to the central effects of leptin on POMC neurons that are also stimulated by estrogen receptor activation [8]. Furthermore, AgRP and NPY expression in the hypothalamus has been shown to change throughout the estrous cycle with decreased food intake during peak estrogen levels [32].

Indeed, females are known to have higher oestrogen levels, and it was reported that it exerts anorexigenic effects by enhancing leptin sensitivity [4, 9]). In particular, 17β-estradiol (E2) enhances POMC neurotransmission to inhibit NPY/AgRP neurons [44].

Growing evidence indicates that HFD consumption induces hypothalamic inflammation accompanied by reactive gliosis [3, 42]. Thus, we aimed to investigate the neuroinflammation profile in our model by performing quantitative real-time PCRs targeting several markers and using immunohistochemistry methods. In contrast to previous studies [3], we did not observe significantly greater mRNA expression levels of proinflammatory cytokines in HFD-fed mice. However, we noted that mRNA transcriffig. 1pt levels for these cytokines in the hypothalamus extracts were often low and below the threshold of experimental detection (Ct > 35) both for SD and HFD mice, thus representing a quantitative bias. Despite that fact, we found that the expression of mRNAs encoding Cd11b and Iba1 was, respectively, higher and lower only in the F-HFD group and differed in a sex-dependent manner. These markers are expressed by both microglia and perivascular myeloid cells as well as infiltrating macrophages. Therefore, these data require careful interpretation. Indeed, nonspecific targeting does not allow us to identify the cell origin of these findings. We could exclude the possibility of potential infiltration of peripheral macrophages because they are not presumed to be present in the healthy CNS, unless an increase in blood‒brain barrier (BBB) permeability occurred in our model. To address this issue, we targeted the purinergic receptor P2RY12, which has been identified as a specific microglial marker allowing to distinguish microglia from macrophages [38, 48]. More precisely, this receptor, expressed on ramified processes, is involved in microglial membrane ruffling and chemotaxis [48]. Thus, its overexpression contributes to process extensions followed by migration, the first step of neuroinflammation [16]. Nevertheless, the *P2ry12* mRNA expression did not differ between the SD and HFD groups in both sexes. However, the decrease in the level of the *Iba1* mRNA in the F-HFD group was confirmed by immunofluorescence staining for the IBA1 protein in the ARC where we also reported a sex effect. These results could be associated with a particular microglial phenotype called “dark” microglia. Indeed, this population is particularly dominant in the hypothalamus and is known to downregulate IBA1 and P2RY12 but strongly expresses CD11b, which is involved in synaptic pruning [45]. Taken together, these findings allowed us to provide better precisions on the immunologically active profile of microglia in female mice after 14 weeks of HFD.

The deleterious effects of chronic HFD feeding on body changes and metabolic disturbances were not concomitant with hypothalamic inflammation, at least from our RT‒qPCR studies, which must be confirmed by further analyses. We hypothesized that neuroinflammation could be more prominent at earlier stages of the exposure to HFD. To address this question, we performed a kinetic to analyse the hypothalamic response during the initial phase of HFD feeding.

In this second cohort, male and female mice were exposed to HFD for 3, 7, 14 or 28 days. As suggested by the first study, male mice responded earlier to HFD exposure in terms of body weight gain (starting from d3) compared to female mice where the response was significant at d14. These differences were evident both in term of absolute weight gain and normalized weight when compared to initial weight, showing that HFD does indeed triggers sex-dependent responses in earlier stage of HFD exposure for these criteria. This delayed response and protection from short-term HFD consumption has already been highlighted in the literature [23]. Indeed, this latter study reported that consumption of a HFD leads to hyperphagia in male rodents. In addition, female mice have greater energy expenditure and are more resistant to body weight gain. Moreover, it has been reported that female mice metabolic response allowed to maintain a normal body weight [8].

Similar to the first cohort (w14), the consumption of a short-term HFD quickly led to a downregulation of *Npy* and *Agrp* (from d3) for both sexes, which was maintained at d28 for male mice. Moreover, we found higher levels in *Pomc* gene expression at d3 and d14 for M- and F-HFD mice, respectively, which is interesting considering that body weight gain almost doubled at this time point compared to the previous measures in this group. This could be a potential compensatory mechanism to restore energy homeostasis and limit weight gain.

Taking into account that some of their data were obtained in rats [47], our RT‒qPCR analysis did not reveal a complex “on–off-on” pattern with significantly elevated hypothalamic levels of *Il6, Il1b, Tnf* or glial markers in HFD mice except to a transient increase of *Il6* levels in M-HFD at d3. However, we systematically reported a sex-dependent response for all studied targets. Especially, the expression profile differences of *Cd11b* and *Fizz1* mRNAs were significant between male and female mice. Taken together, these results only partially support the establishment of anti-inflammatory mechanisms in response to HFD.

Hypertrophic and hyperplasic astrocytes are concurrently observed in the hypothalamus even 1 day after HFD feeding to reduce lipid overload [47]. Since GFAP is the hallmark intermediate filament, we expected to observe an increase in *Gfap* mRNA expression in HFD mice compared to that in SD groups. In contrast, we observed a trend toward downregulation during this kinetic which significantly differed by the gender. We could suppose a variability in expression is linked to the growth of mice or reflects a decrease in the size, number, and/or fibrous character of astrocytes. Immunostaining aimed to clarify these observations. In the same way that for the first study, we performed immunofluorescence staining within the ARC due to its proximity to the median eminence (ME), where the fenestrated vascular endothelium lacks a BBB. Indeed, among hypothalamic regions, the ARC provides a positional advantage to rapidly sense excessive fatty acids from the diet. Interestingly, we anew found time effect of the GFAP + cell number but no significant difference between male and female mice. However, a significant sex difference was observed of the number of IBA1-positive cells. Especially, F-HFD displayed less of IBA1 + cells compared to M-HFD at d14, a result already observed at w14.

The classical markers, such as increased expression of *Iba1* and *Gfap* or various cytokines, provide only indirect information about neuroinflammation and are insufficient to assess this process. Therefore, a detailed morphological analysis of glial cells was performed in order to provide additional and critical insights into hypothalamic adaptations under HFD. By studying multiple parameters, we reported sex-dependent responses. Regarding Sholl analysis profiles of microglia, M-HFD mice continued to display a shift towards reduced complexity level compared to M-SD at w14, whereas this shift was only present at d28 for F-HFD. Sholl analysis of astrocytes revealed a new earlier response at d28 in F-HFD with greater complexity than in M-HFD mice. These results were concomitant with other parameters analysed such as more branch/terminal points as well as higher branch level and filament length in the F-HFD compared to M-HFD mice at d28, mostly. Astrocytes along with microglia are highly dynamic cells and display a complex spectrum of morphotypes depending on the biological context as well as the local environment [34]. The more pronounced effects on astrocyte morphology than on microglia are consistent with their involvement in several metabolic processes (glucose homeostasis maintenance, insulin and leptin sensing, etc.) [15]. Moreover, immunofluorescence assays have recently shown that the microglial signature varies according to hypothalamic nuclei and dietary fat content, particularly in mice [30]. Astrocytes are the main site of fatty acid metabolism and oxidation in the brain [8]. Short chain fatty acids in HFD may be responsible for astrocytic reactivity. Indeed, it has been reported that HFD increases circulating palmitic acid levels, as well as central levels, and can directly activate astrocytes in vitro [18]. Taken together with the mRNA expression analysis, these changes in astrocytic morphology (and supposedly activity) might be interpreted as metabolic adaptations to reduce lipid overload and restore homeostasis rather than an inflammatory response. Still, we observed a reduction in the complexity of microglial ramification which translate to a reactive phenotype. HFD has been showed to trigger neuroinflammation and to induce a morphological transition from a highly ramified to a deramified state, retracting their fine processes while simultaneously undergoing somatic hypertrophy [38]. In this last experiment, we reported differences in glial parameters under HFD conditions in a time and sex dependent manner, but it remains unclear whether these changes translate to proinflammatory and/or neuroprotective mechanisms or altered glial function.

This study demonstrates that a strong inflammatory hypothalamic profile is not required to induce obesity in mice and questions the role of the modulation of glial cell function in the induction of HFD phenotype. Therefore, further investigation is needed to better characterize this relationship. This could be achieved using a multidimensional integration of transcriptomic, metabolomic, proteomic and epigenetic approaches.

Because mice were co-housed (4 animals per cage), we were not able to measure neither locomotor activity nor energy expenditure for each animal. However, *Crh* mRNA levels only decreased in the M-HFD group, which is known to increase energy expenditure [28]. Similar studies assessing these parameters have shown divergent findings. Indeed, some data suggest that females are more active than males but in rats [14]. Studies carried out in C57Bl/6J mice reported that male mice reduced physical activity in response to a HFD with unaffected food intake, while female mice showed increased food intake without changes in physical activity and exhibited increased energy expenditure. Some studies did not show sex differences in locomotor activity, which is also not affected by HFD [29]. This variability between studies can be linked to inconsistencies in study designs using a variety of diet types, fat content with different ω6/ω3 ratios [40], onset of intervention and duration of diet, making elucidating what variables cause the observed divergences in the literature challenging. Monitoring of daytime locomotor activity and respiratory exchanges by indirect calorimetry could, in a future study, shed more light on these present results at the metabolic level. In addition, our study does not provide data regarding neuronal protein expression or neuronal-glial cross talk. Studies have shown that changes in astrocytic morphology induced by HFD can influence glutamate clearance as well as the induction of long-term potentiation in the hippocampus [36]. In the ARC, studies have revealed that HFD can induce an increased ensheathment of POMC neurons perikarya, thus increasing their excitatory tone [21]. Interestingly, food deprivation induces similar changes in AgRP neurons through GABA-dependent mechanisms, thus increasing their excitability [51]. Given the rapid expression in HFD-fed animals of the neuronal plasticity marker PSA-NCAM, which promotes synaptic reorganization by weakening cell-to-cell interactions, we can hypothesize that the modifications of glial cell morphology observed in our model likely affect neuronal function and plasticity [2]. This plasticity seems to act as a mechanism limiting the weight gain induced by the HFD. To test this, electrophysiological recordings of arcuate nucleus neurons would be required to examine synaptic transmission in both sexes or immunohistochemistry experiments targeting neuronal plasticity markers on POMC/AgRP neurons could be performed. Despite these limitations, our study emphasizes the crucial role of carefully examining sex differences and timing in DIO models.

## Conclusion

In summary, the present study provides nuanced data regarding hypothalamic inflammation under HFD conditions. However, we support evident sex differences in response to short- and long-term HFD consumption, suggesting that the prevention and treatment of obesity should be considered in a sex-dependent manner.

## Supplementary Information

Additional file 1: Fig. S1: Visualization of glial cells by immunofluorescence in ARC after 14 weeks of SD or HFD. These representative images were taken within the ARC and show GFAP and IBA1 immunopositive cells in male (top panel) and female mice (bottom panel) after 14 weeks of SD or HFD feeding. Scale bar: 50 µm. 3V: third ventricle.

Additional file 2: Fig. S2: Weekly monitoring of food consumption in male and female mice over 12 weeks of diet. Average estimation of food consumed in grams and kilocalories (g and kcals) per day and per animal fed either SD or HFD in male and female mice. N=6 cages/group, total of grams and kcals measured per cage were divided by the number of mice per cage (n=4/cage). Average values per cage were compared with 3-way repeated measure ANOVA followed by Tukey post hoc tests (*p<0.05 male SD *vs* HFD; #p<0.05 female SD *vs* HFD; α p<0.05 male HFD *vs* female HFD). Data are presented as mean ± standard error of the mean (SEM). Male and female mice fed HFD per day ingested in average 3.0±0.03 g (15.73±0.16 kcals) and 2.4±0.02 g (12.71±0.11 kcals) compared to male and female mice fed with SD which per day consumed in average 4.1±0.04 g (13.69±0.13 kcals) and 3.6±0.05 g (12.16±0.17 kcals), respectively.

Additional file 3: Table S1: Composition of the high fat diet.

Additional file 4: Table S2: Primer sequences used for RT-qPCR.

Additional file 5: Table S3: Summary of statistical tests.

## Data Availability

No datasets were generated or analysed during the current study.

## Abbreviations

AgRP: Agouti-related protein
ARC: Arcuate nucleus
BDNF: Brain-derived neurotrophic factor
CRH: Corticotropin-releasing hormone
DIO: Diet-induced obesity
GFAP: Glial fibrillary acidic protein
HFD: High-fat diet
Iba1: Ionized calcium-binding adapter molecule 1
IL: Interleukin
iNOS: Inducible nitric oxide synthase
MC4R: Melanocortin-4 receptor
NPY: Neuropeptide Y
PBS: Phosphate-buffered saline
PFA: Paraformaldehyde
POMC: Proopiomelanocortin
P2RY12: Purinergic receptor P2Y12
ROI: Region of interest
RT-qPCR: Reverse transcriptase quantitative polymerase chain reaction
SD: Standard diet
TNF: Tumor necrosis factor
