# Supplementary figures and images for: Sex-dependent effects of a high-fat diet on the hypothalamic response in mice
Source: Biol Sex Differ. 2025 Feb 25;16:17. doi: 10.1186/s13293-025-00699-3 (PMC11854408; doi:10.1186/s13293-025-00699-3)

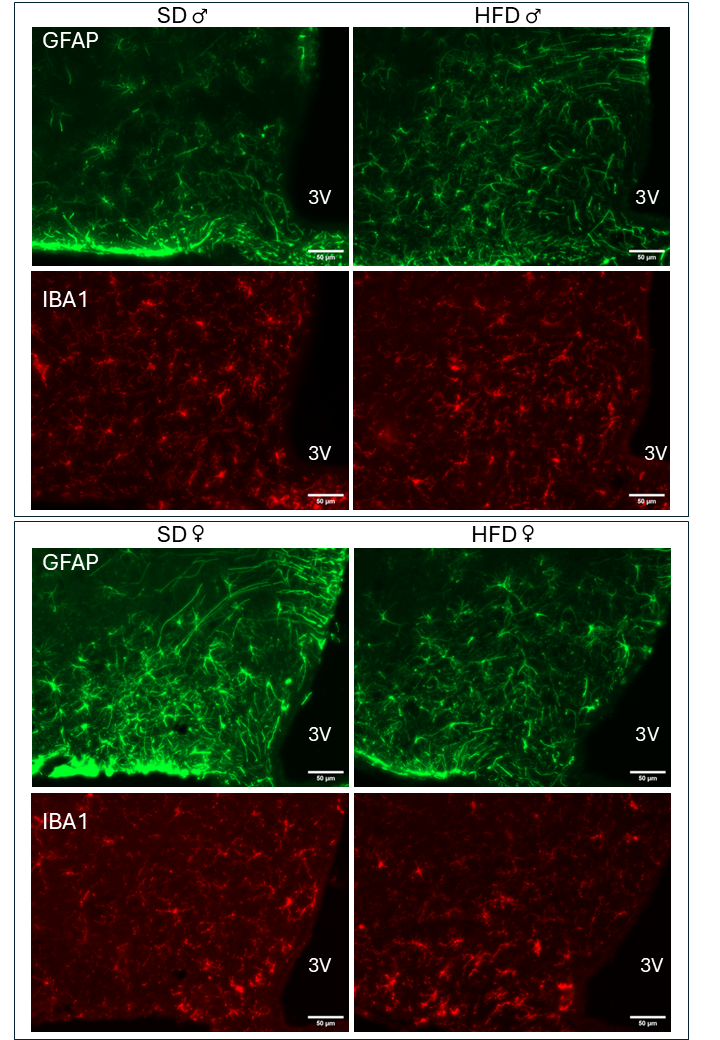

Supplement: Supplementary file 1 — Additional file 1: Fig. S1: Visualization of glial cells by immunofluorescence in ARC after 14 weeks of SD or HFD. These representative images were taken within the ARC and show GFAP and IBA1 immunopositive cells in male (top panel) and female mice (bottom panel) after 14 weeks of SD or HFD feeding. Scale bar: 50 µm. 3V: third ventricle. [file 13293_2025_699_MOESM1_ESM.tif]

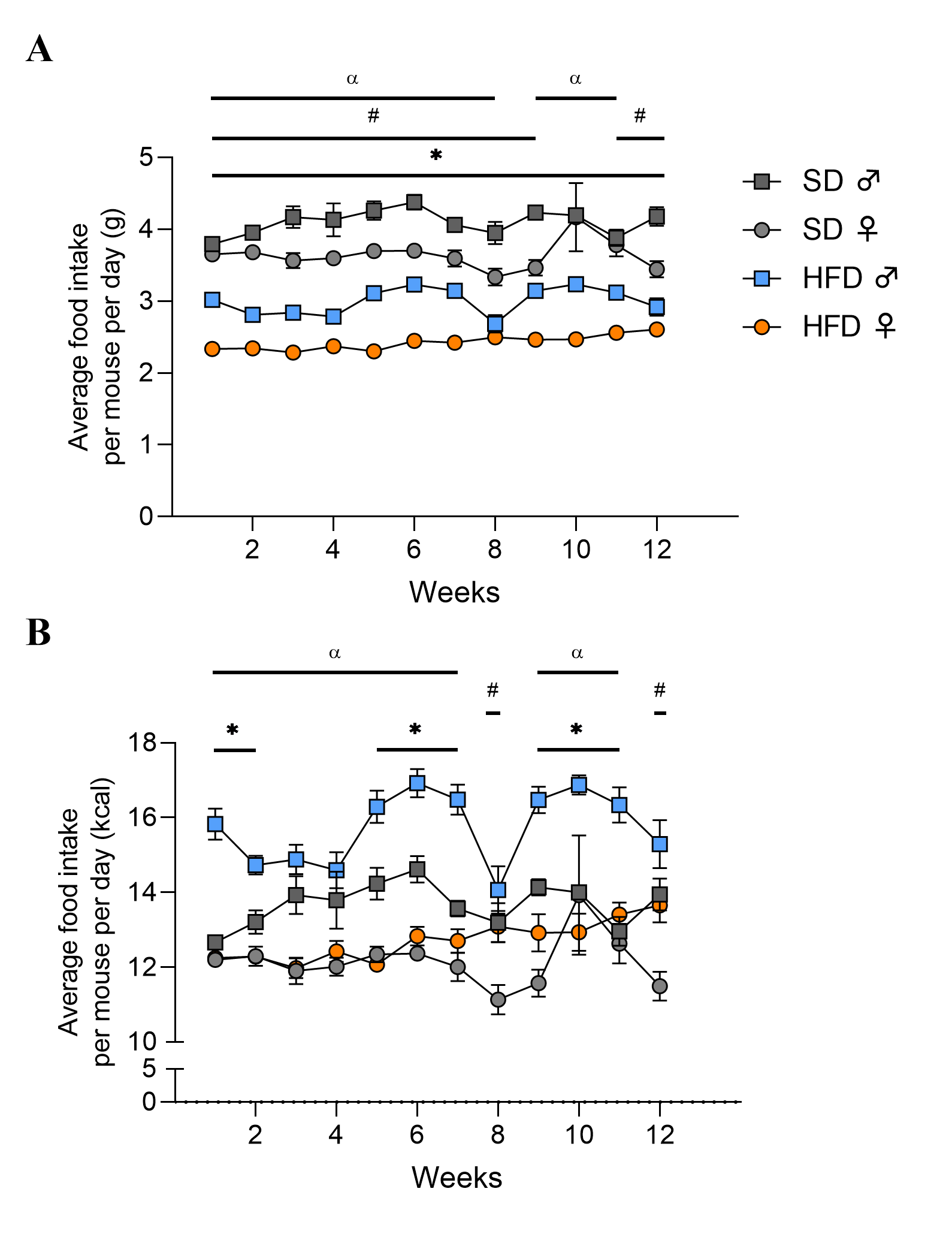

Supplement: Supplementary file 2 — Additional file 2: Fig. S2: Weekly monitoring of food consumption in male and female mice over 12 weeks of diet. Average estimation of food consumed in grams and kilocalories (g and kcals) per day and per animal fed either SD or HFD in male and female mice. N=6 cages/group, total of grams and kcals measured per cage were divided by the number of mice per cage (n=4/cage). Average values per cage were compared with 3-way repeated measure ANOVA followed by Tukey post hoc tests (*p<0.05 male SD vs HFD; #p<0.05 female SD vs HFD; α p<0.05 male HFD vs female HFD). Data are presented as mean ± standard error of the mean (SEM). Male and female mice fed HFD per day ingested in average 3.0±0.03 g (15.73±0.16 kcals) and 2.4±0.02 g (12.71±0.11 kcals) compared to male and female mice fed with SD which per day consumed in average 4.1±0.04 g (13.69±0.13 kcals) and 3.6±0.05 g (12.16±0.17 kcals), respectively. [file 13293_2025_699_MOESM2_ESM.tif]
