# Supplementary material for: Sex-dependent effects of a high-fat diet on the hypothalamic response in mice
Source: Biol Sex Differ. 2025 Feb 25;16:17. doi: 10.1186/s13293-025-00699-3 (PMC11854408; doi:10.1186/s13293-025-00699-3)
Supplement: Supplementary file 4 — Additional file 4: Table S2: Primer sequences used for RT-qPCR. [file 13293_2025_699_MOESM4_ESM.docx]

**Supplementary Table S2: Primers used for real time PCR.**

| Gene  names | Forward primer (5’🡪3’) | Reverse primer (5’🡪3’) | Tm uses (°C) |
| --- | --- | --- | --- |
| *Gapdh* | CATCACTGCCACCCAGAAGA | AAGTCGCAGGAGACAACfiCT | 60.0 |
| *Actb* | CTCTGGCTCCTAGCACCATGAAG | GTAAAACGCAGCTCAGTAACAGTCCG | 64.0 |
| *Il1b* | CCCAAAAGATGAAGGGCTGC | AAGGTCCACGGGAAAGACAC | 64.0 |
| *Il6* | CACTTCACAAGTCGGAGGCT | CTGCAAGTGCATCATCGTTGT | 62.0 |
| *Tnf* | TGTCTACTCCTCAGAGCCCC | TGAGTCCTTGATGGTGGTGC | 65.0 |
| *iba1* | ATCAACAAGCAATTCCTCGATGA | CAGCATTCGCTTCAAGGACAT | 61.0 |
| *Gfap* | TGGAACAGCAAAACAAGGCG | CTGTCTATACGCAGCCAGGT | 60.0 |
| *P2ry12* | ACGGACACTTTCCCGTATCC | AAGTTCCCAAAGCCCTCTGT | 60.0 |
| *Npy* | CTGCGACACTACATCAATCT | CTTCAAGCCTTGTTCTGG | 56.0 |
| *Pomc* | CCTCCTGCTTCAGACCTCCA | GGCTGTTCATCTCCGTTGC | 62.0 |
| *Agrp* | ACTGAAGGGCATCAGAAGGC | TTGAAGAAGCGGCAGTAGCA | 60.0 |
| *Cd11b (Itgam)* | ATGGACGCTGATGGCAATACC | TCCCCATTCACGTCTCCCA | 62.0 |
| *Fizz1* | CCTGAGATTCTGCCCCAGGAT | TTCACTGGGACCATCAGCTGG | 60.0 |
| *Nos2* | CCGAAGCAAACATCACATTCA | GGTCTAAAGGCTCCGGGCT | 60.0 |
| *Arg1* | GTTCCCAGATGTACCAGGATTC | CGATGTCTTTGGCAGATATGC | 60.0 |
| *Mc4r* | TCTCTATGTCCACATGTTCCTG | GGGGCCCAGCAGACAACAAAG | 62.0 |
| *Crh* | TAAAGAAAATGTGGCCCCAAGG | CTTCCACTGCAGCTCCAAATAA | 60.0 |
| *Bdnf* | TGTGACAGTATTAGCGAGTGGGT | TACGATTGGGTAGTTCGGCATT | 66.0 |
